# The role of neurovascular coupling dysfunction in cognitive decline of diabetes patients

**DOI:** 10.3389/fnins.2024.1375908

**Published:** 2024-03-21

**Authors:** Lin Feng, Ling Gao

**Affiliations:** Department of Endocrinology, Renmin Hospital of Wuhan University, Wuhan, China

**Keywords:** diabetes, cognitive function, neurovascular coupling, functional imaging, treatment

## Abstract

Neurovascular coupling (NVC) is an important mechanism to ensure adequate blood supply to active neurons in the brain. NVC damage can lead to chronic impairment of neuronal function. Diabetes is characterized by high blood sugar and is considered an important risk factor for cognitive impairment. In this review, we provide fMRI evidence of NVC damage in diabetic patients with cognitive decline. Combined with the exploration of the major mechanisms and signaling pathways of NVC, we discuss the effects of chronic hyperglycemia on the cellular structure of NVC signaling, including key receptors, ion channels, and intercellular connections. Studying these diabetes-related changes in cell structure will help us understand the underlying causes behind diabetes-induced NVC damage and early cognitive decline, ultimately helping to identify the most effective drug targets for treatment.

## 1 Introduction

**FIGURE 1:**
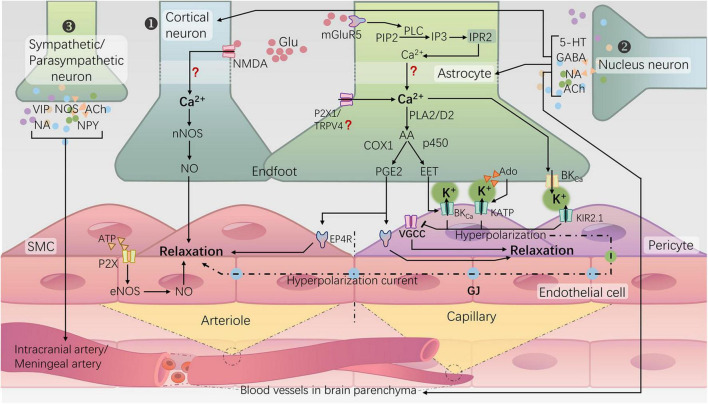
Neurovascular regulation mechanisms for cerebral blood flow in the brain and NVC. ① Rapid responsive cortical NVC pathway (classical NVC pathway): a. Neurons induce arteriolar dilation through the N-methyl-D-aspartate receptor (NMDA)–neuronal nitric oxide synthase (nNOS) pathway, leading to the production of NO; b. Astrocytes, via the activation of metabotropic glutamate receptor 5 (mGluR5), initiate a cascade reaction that releases Ca2+ from the endoplasmic reticulum. Subsequently, the rise in intracellular Ca2+ in the endfeet activates phospholipase A2 (PLA2) or phospholipase D2 (PLD2), ultimately generating prostaglandin E2 (PGE2) and epoxyeicosatrienoic acids (EETs). Additionally, activation of potassium channels, including large-conductance calcium-activated potassium channels (BKCa), ATP-sensitive potassium channels (KATP), and inward rectifying potassium channels 2.1 (KIR2.1), on the vascular wall leads to hyperpolarization and relaxation of arterioles and capillaries. ② Subcortical-nuclei-neurotransmitter regulatory pathways: many subcortical nuclei, such as the locus coeruleus, basal ganglia, and raphe nucleus, modulate cortical blood flow directly or indirectly through the release of neurotransmitters such as norepinephrine (NA) (Bekar et al., 2012), acetylcholine (ACh) (Lecrux et al., 2017), gamma-aminobutyric acid (GABA) (Kocharyan et al., 2008), and serotonin (5-HT) (Cui et al., 2013) within the cortex, involving interactions with cortical neurons or astrocytes. ③ Peripheral sympathetic/parasympathetic postganglionic neuron regulatory pathways: the sympathetic nerves originating from the superior cervical ganglion, through the release of norepinephrine (NA) and neuropeptide Y (NPY), induce constriction of the major cerebral arteries. Conversely, parasympathetic nerves originating from the sphenopalatine ganglion release acetylcholine (ACh), vasoactive intestinal peptide (VIP), and nitric oxide synthase (NOS) substances, exerting a vasodilatory effect (Hamel, 2006; Seifert and Secher, 2011).

The brain, as the highest level of the nervous system, serves as the control center for human behavior and cognitive functions. It is also the most energy-consuming organ in the human body. In which neurons exhibit a high metabolic rate but lack energy reserves (Raut et al., 2023). To ensure an adequate energy supply for neurons, the brain has evolved a mechanism to regulate local cerebral blood flow (CBF), known as NVC or functional hyperemia. This mechanism involves dynamic changes in local blood flow supply in response to the electrical activity of neurons to meet metabolic demands. There are three types of neurovascular regulation mechanisms for CBF in the brain (Figure 1): (1) Cortical neuron neurotransmitter regulation mechanism (i.e., the classic NVC pathway); (2) Subcortical nucleus-neurotransmitter regulation mechanism (Kocharyan et al., 2008; Bekar et al., 2012; Cui et al., 2013; Lecrux et al., 2017); (3) Regulation mechanism of peripheral sympathetic/parasympathetic postganglionic neurons vasoactive substances (Hamel, 2006; Seifert and Secher, 2011). The classic NVC pathway relies on the neurovascular unit (NVU), which involves the transmission of information among neurons, astrocytes, endothelial cells, smooth muscle cells (SMCs), and pericytes (Schaeffer and Iadecola, 2021). The generation of neuronal action potentials serves as the initiating factor, with astrocytes sensing neuronal activity and their endfeet directly connecting to blood vessels, facilitating the transmission of neuronal activity signals to the local vascular system (Sweeney et al., 2016; McConnell et al., 2017). The SMCs of arterioles and pericytes of capillaries, serving as effectors in NVC, receive signals from the aforementioned cells to regulate vascular tone (Stackhouse and Mishra, 2021). Any damage to any component of the NVU can lead to functional impairment of NVC, resulting in a mismatch between CBF supply and neuronal activity. This, in turn, leads to chronic damage to brain neurons and a decline in cognitive function (Iadecola, 2017; Turner, 2021).

Diabetes mellitus (DM), characterized by high blood sugar levels, is considered a significant risk factor for cognitive impairment (Roberts et al., 2014; Koekkoek et al., 2015). Previous studies have shown that regional cerebral perfusion in patients with type 2 diabetes mellitus (T2DM) is significantly reduced in multiple locations (including the occipital lobe, regions involved in the default mode network, and cerebellum). Moreover, this reduction is associated with widespread cognitive decline (including impairments in learning, memory, attention, and executive function) (Cui et al., 2017; Bangen et al., 2018; Wang et al., 2021; Liu et al., 2022). However, there were no significant differences in total CBF between the T2DM group and the healthy group (Tiehuis et al., 2008; Brundel et al., 2012). This observation may suggest impaired local regulation of CBF in T2DM, which could contribute to cognitive decline (Mogi and Horiuchi, 2011; Duarte et al., 2015; Lourenço et al., 2017; Shekhar et al., 2017). Given the crucial role of NVC in regulating local CBF, its relationship with T2DM-related cognitive impairment is increasingly being recognized.

In this review, we initiate our exploration from neuroimaging studies of diabetes-associated cognitive decline, focusing on the role of impaired NVC, also known as neurovascular uncoupling, in the context of cognitive deterioration in diabetes. Our emphasis will be on investigating the potential mechanisms through which diabetes induces NVC impairment and on outlining future research directions. By doing so, we aim to provide insights into the diagnosis and treatment strategies for early cognitive decline associated with diabetes.

## 2 Evidence of cognitive decline in diabetes patients

Diabetes is a risk factor for cognitive decline. Moreover, as the disease progresses, complications emerge, and blood glucose control deteriorates, the risk of developing cognitive impairment increases (Dao et al., 2023; Sakib et al., 2023). Cognitive impairment, in turn, negatively impacts patients’ self-care and blood glucose management, fostering a vicious cycle (Sinclair and Abdelhafiz, 2020). Patients with T2DM experience varying degrees of decline in executive function, memory, and information-processing abilities (Sadanand et al., 2016; Wang H. et al., 2023). These cognitive deficits are closely associated with changes in brain function and structure related to T2DM, such as cerebral perfusion deficits (Cui et al., 2017; Wang et al., 2021), brain white matter damage (Jing et al., 2022; Liu et al., 2024), and hippocampal volume atrophy (Hirabayashi et al., 2016; Ohara et al., 2020; Zhang W. et al., 2021).

As mentioned earlier, CBF significantly decreases in multiple regions in patients with T2DM (Cui et al., 2017; Bangen et al., 2018; Wang et al., 2021; Liu et al., 2022). Endothelial dysfunction and reduced CBF are considered early changes preceding the occurrence of cognitive deficits (Sadanand et al., 2016; Gorelick et al., 2017; Wang H. et al., 2023). Imbalances in endothelium-derived vasoconstrictors and vasodilators can lead to cerebrovascular dysfunction, which may result in CBF dysregulation, with the endothelium being considered an early target for metabolic diseases, including diabetes (Kiss et al., 2020b). Early studies found decreased levels of Sirtuin1 (SIRT1) in the aorta of diabetic mice compared to normal controls (Zhou et al., 2011). Further research revealed that endothelial-specific overexpression of SIRT1 in diabetic transgenic mice compared to diabetic wild-type mice would decrease levels of aging markers such as p53, p21, PAI-1, and p66Shc in the aorta (Chen et al., 2012). Senthil et al. (2017) study indicated that activation of nuclear factor erythroid 2-related factor 2 (Nrf2)-mediated antioxidant genes could prevent high glucose-induced endothelial aging and apoptosis (Senthil et al., 2017). High glucose conditions induce premature endothelial cell aging, leading to CBF dysregulation (Biessels and Despa, 2018; Prattichizzo et al., 2018; Balasubramanian et al., 2021). A recent review (Hwang et al., 2022) extensively summarized the molecular pathways related to endothelial cell aging. Understanding these diabetes-related changes in pathways is of significant importance for improving endothelial dysfunction and CBF dysregulation. Additionally, Li Y. et al. (2018) recently found that cognitive impairment and CBF reduction in T2DM mice may be associated with the RhoA/ROCK/moesin and Src signaling pathways (Li Y. et al., 2018). Castaneda-Vega et al. (2023) identified the role of brain vascular inhibitory G protein-coupled receptor signaling in maintaining CBF, which may be useful for developing new drug treatment approaches for preventing and treating cerebrovascular dysfunction (Castaneda-Vega et al., 2023).

Numerous clinical studies have found a close relationship between white matter injury and cognitive function (especially executive function) deterioration (Fletcher et al., 2018; Yamanaka et al., 2019; Jeong et al., 2022; Scamarcia et al., 2022; Wang et al., 2022; Dewenter et al., 2023). Simultaneously, researchers have observed abnormal changes in the macrostructure of white matter, including larger volumes of white matter lesions (WMLs), more white matter hyperintensities, and abnormal white matter network connectivity, in prediabetic (Jing et al., 2022) and diabetic patients (Sanahuja et al., 2016; Xiong et al., 2019; Huang et al., 2021). Moreover, these changes are associated with cognitive decline (Jing et al., 2022; Liu et al., 2024). It is widely believed that white matter injury in patients with DM is related to an increased burden of small vessel disease (Novak et al., 2006; Schneider et al., 2017; Georgakis et al., 2021). White matter consists of nerve axons and glial cells supporting the axons, such as oligodendrocytes. Studies have indicated that in the process of WMLs after chronic ischemia in diabetic patients, the proliferation and survival of oligodendrocyte progenitor cells (OPCs) may play an important role (Yatomi et al., 2015; Ma et al., 2018), and inhibiting Na+-K+-Cl- cotransporter 1 can significantly improve white matter injury and cognitive impairment caused by chronic cerebral hypoperfusion by enhancing OPCs proliferation (Yu et al., 2018). Additionally, high glucose concentrations may lead to polarization of microglia/macrophages toward a pro-inflammatory phenotype, severely affecting oligodendrocyte differentiation and white matter repair (Ma et al., 2018). Despite incomplete understanding of the molecular mechanisms, abnormalities in white matter microstructure are still considered an important biomarker and a cause of diabetes-induced neurological disorders (Jing et al., 2022; Liu et al., 2024).

As is well known, the hippocampus is closely associated with learning and long-term memory functions. Imaging studies have suggested that middle-aged and elderly patients with T2DM exhibit more extensive hippocampal atrophy compared to control groups (Hirabayashi et al., 2016; Ohara et al., 2020; Zhang W. et al., 2021). Interestingly, Zhang W. et al.’s (2021) findings indicate that in middle-aged T2DM patients, hippocampal atrophy is more strongly correlated with cognitive impairment than microvascular lesions. Due to differences in microvasculature, the NVC in the hippocampus is weaker than in the neocortex (Shaw et al., 2021). When pathological factors (such as a high glucose environment) impair NVC, the hippocampus is more susceptible to hypoxic damage, leading to hippocampal atrophy (Shaw et al., 2021; Zhang et al., 2022).

Animal studies suggest that diabetes leads to a decrease in the number of active neurons in the hippocampal region, possibly due to reduced neural stem cell proliferation and differentiation (Hwang et al., 2008, 2010; Ho et al., 2015), hippocampal cell aging (Wu et al., 2019) and increased apoptosis (Yan et al., 2019). In the hippocampus of diabetic rats, early mechanistic studies have reported impaired protein transport from the soma to dendrites (Gaspar et al., 2010), synaptic vesicle depletion (Magariños and McEwen, 2000), and altered neurotransmitter release (Misumi et al., 2008; Satoh and Takahashi, 2008). These diabetes-related effects may also contribute to the development of cognitive decline (Gaspar et al., 2010). Recently, Xiang et al. (2024) conducted single-cell RNA sequencing of the hippocampus in db-/- diabetic mice and found upregulation of genes involved in neuroactive ligand-receptor interaction, nervous system development, and inflammatory processes in the cognitive impairment group compared to the normal control group. Among them, the cross-gene Sstr2 may play an important role in regulating synaptic plasticity (Xiang et al., 2024). Research by Burillo et al. (2021) suggests that the harmful accumulation of amylin protein (a pancreatic secretory amyloid-like protein hormone) in pancreatic β cells may cause damage through the release of exosomes, which may be captured by hippocampal cells via endocytosis mechanisms, resulting in damage. Meanwhile, accumulation of amylin in the blood and brain microvasculature can lead to cerebral microbleeds, decreased CBF, white matter ischemia, and neurofunctional deficits. This is believed to cause oxidative damage to cell membrane lipids and activation of pro-inflammatory signaling pathways, leading to macrophage activation and vascular infiltration (Ly et al., 2017; Despa and Goldstein, 2021). Although cognitive decline in T2DM patients is associated with the aforementioned changes in brain function and structure, specific molecular mechanisms and effective preventive and therapeutic measures require further research and exploration.

## 3 Clinical evidence of NVC impairment in diabetes patients

Technic used for NVC measurement: The NVC mechanism also forms the physiological basis for blood oxygenation level-dependent (BOLD) functional imaging techniques of the brain, including functional magnetic resonance imaging (fMRI) and functional near-infrared spectroscopy (fNIRS) (Howarth et al., 2021). These imaging techniques monitor changes in the concentrations of oxygenated and deoxygenated hemoglobin in response to increased local CBF caused by neuronal electrical activity. By observing the relative changes in hemoglobin concentration, these imaging techniques allow for the examination of alterations in neuronal activity. The combination of functional imaging techniques with arterial spin labeling (ASL) MRI, which reflects cerebral tissue perfusion, enables the non-invasive measurement of the NVC status in the human brain under disease conditions (Howarth et al., 2021).

Hu et al. (2019) used fMRI and ASL to measure NVC in the brains of age-matched T2DM patients and healthy controls. They found that T2DM patients exhibited significantly lower NVC in nearly all brain regions. Specifically, lower NVC in the left hippocampus and amygdala was significantly correlated with poorer performance on the Stroop Color-Word Test, which reflects inhibitory functions in executive function (Okayasu et al., 2023). Yu et al. (2019) further confirmed the presence of NVC impairment in early-stage T2DM patients and established a correlation between NVC impairment and decline in executive function, with improved executive function performance as NVC improves (Yu et al., 2019). They suggest that NVC dysfunction is one of the potential mechanisms underlying mild cognitive impairment (MCI) associated with T2DM. Yu et al. (2019) also found that certain NVC parameters could serve as biomarkers for early assessment of cognitive decline in T2DM patients, which also contribute to a better understanding of NVC mechanisms (Ni et al., 2023). Additionally, three other clinical studies have identified changes in NVC during the early stages of diabetes (Duarte et al., 2015, 2023; Monteiro et al., 2021). These findings collectively validate previous conclusions that neurovascular uncoupling occurs in the early stages of T2DM and promotes the transition from diabetes-related mild cognitive impairment to dementia (Mogi and Horiuchi, 2011; Duarte et al., 2015; Venkat et al., 2016; Shekhar et al., 2017). T2DM patients without mild cognitive impairment are considered the best target population for preventive interventions (Yu et al., 2019; Kovacs-Oller et al., 2020; Ni et al., 2023). Furthermore, a longitudinal study by Zhang Y. et al. (2021) over 5 years indicated that T2DM may accelerate NVC damage in specific brain regions (left insula), leading to memory decline (Zhang Y. et al., 2021). Canna et al. (2022) identified spatial patterns of decreased NVC in the default mode network of T2DM patients, accompanied by isolated increases in NVC in the dorsal attention network (DAN) and ventral attention network (VAN), with DAN and VAN NVC abnormalities associated with declines in visual-spatial cognitive abilities (Canna et al., 2022). This may reflect the emergence of compensatory processes in response to changes in neurovascular status in T2DM patients. While these clinical studies have revealed the association between diabetes and NVC impairment, the mechanisms underlying diabetes-induced NVC disruption remain unclear, and targeted diagnostic and preventive interventions lack theoretical foundations and direction.

The comparison of fMRI vs. fNIRS on NVC functional evaluation: Current fMRI studies have certain limitations. Most studies focus on detecting the state of neuronal activity and CBF in the resting state of the brain. In reality, the energy demands of the brain are higher during cognitive tasks than during rest. Therefore, observations made in the resting state may only reflect a partial understanding of impaired NVC in diabetes patients. In comparison to fMRI, functional near-infrared spectroscopy (fNIRS) can be conducted in a more real-life environment, making it easier to observe and record cortical activity during cognitive tasks (Mazaika et al., 2020). Tamas’s review discusses the potential applications of fNIRS-based methods in studying NVC responses (Mazaika et al., 2020). However, research on diabetes-related NVC changes using fNIRS is relatively sparse at the moment, making it a fertile area for future investigation.

## 4 Hyperglycemic and NVC impairment

The characteristics of diabetes include impaired glucose metabolism and a hyperglycemic environment resulting from insulin resistance and/or deficiency. High blood sugar is the initial pathological factor in diabetes complications, leading to cellular damage in the brain by elevating glucose levels. Dorner et al. (2003) utilized a retinal vessel analyzer to measure the retinal vessel diameter, observing a significant reduction in the responsiveness of retinal vessels to flickering light stimuli in healthy young males during acute hyperglycemia (Dorner et al., 2003). Vetri et al. (2017) employed a closed cranial window technique and found that the reactivity of the pial arterioles in the somatosensory cortex decreased in response to sciatic nerve stimulation in type 1 diabetic mice (Vetri et al., 2017). Chhabria et al. (2020) established a zebrafish NVC model, combined with light sheet microscopy, revealing that prolonged exposure to high glucose levels damages NVC in zebrafish (Chhabria et al., 2020). In summary, both acute and chronic hyperglycemia can impair NVC.

The implementation of NVC relies on the NVU. Currently, studies have confirmed that certain components of the NVU exhibit abnormal morphology and function under diabetic conditions (Hayden, 2019; Yan et al., 2020; Little et al., 2022). Oxidative stress is considered one of the pathological mechanisms by which diabetes damages the NVU (Li et al., 2021), including increased levels of reactive oxygen species (ROS) (Jha et al., 2018; Li et al., 2023), the generation of advanced glycation end-products (AGEs) (Yamagishi et al., 2017; Li Y. et al., 2018), abnormal transcriptional activation of nuclear factor kappa B (NF-κB) (Homme et al., 2021), and excessive activation of nicotinamide adenine dinucleotide phosphate (NADPH) oxidase 2 (Rezende et al., 2018; Moon, 2023), among others. Additionally, chronic inflammation is recognized as an important feature of the pathophysiology of central nervous system diseases related to diabetes and is well demonstrated in diabetes experimental models (Robb et al., 2020). Among these, microglia play a role in the invasive destruction within the NVU and are associated with dysfunction of astrocytes (Hayden, 2019). In diabetic animal models, a shift from the M1/M2 polarization phenotype of microglia toward the M1 phenotype is detected in the cortex and hypothalamus, leading to excessive secretion of inflammatory cytokines, which is associated with downregulation of miR-146a expression under high glucose and glucose fluctuations (Huang et al., 2019). Indeed, the mechanisms underlying NVU damage are multifactorial and complex, and some review articles have summarized changes in the NVU under diabetic conditions and their mechanisms (Yan et al., 2020). Based on the current known mechanisms, from the perspective of understanding the implementation process of NVC, diabetes may damage NVC through two main aspects: disrupting key normal structures of neurovascular units and interfering with the transmission of cell signals related to NVC.

### 4.1 Damage to the normal structure of neurovascular units

The end feet structure and astrocytes activation: Astrocytes’ end feet directly wrap around cerebral arterioles and capillaries, undertaking the crucial task of transmitting neuronal activity signals to the local vascular system, which is essential for NVC. SMCs in arterioles are sensitive to potassium concentrations around the vascular wall, and in NVC, astrocytes can mediate vasodilation by momentarily elevating the K+ concentration in the space between their end feet and the blood vessel (Longden and Nelson, 2015; Li and Yang, 2023). Under normal circumstances, the space between astrocyte end feet and blood vessels is extremely narrow (basement membrane width, approximately 20 nm), a critical factor enabling the rapid alteration of potassium concentration around the vascular wall (Longden and Nelson, 2015). However, in diabetic mice, the end feet of astrocytes are separated from the blood vessel wall, which may impact the aforementioned process and is considered an expression of reactive astrocytes (Mauricio et al., 2023).

When the brain is subjected to pathological stimuli, astrocytes are activated and become reactive astrocytes. Transient reactive astrocytes are believed to have a neuroprotective effect (Lorenzo et al., 2021), while persistent hyperglycemia and AGEs mediate sustained activation of astrocytes (Meng et al., 2023), leading to cellular dysfunction and neuronal inflammatory responses. Astrocytes take up glucose through glucose-dependent glucose transporter 1 (GLUT1) on the cell membrane (Maurer et al., 2006). High blood glucose can significantly downregulate the expression of GLUT1 in astrocytes (Shi et al., 2020), resulting in energy and metabolic disturbances. It can also inhibit the migration and proliferation of astrocytes by suppressing the expression of cell cycle proteins D1 and D3 (Li W. et al., 2018). *In vitro* studies have found that high blood glucose increases extracellular ROS levels in cells, mediating the activation of astrocytes through the MEK/ERK1/2 pathway and downstream transcription factors NF-κB and c-Fos/activating protein 1 (AP-1) (Yang et al., 2017). Additionally, elevated AGEs increase the expression of the receptor for AGEs (RAGE) in astrocytes (Han et al., 2014; Nardin et al., 2016), and the interaction between AGEs and RAGE can activate NF-κB through various signaling pathways, such as PI3K/AKT, MEK/ERK1/2 and NADPH oxidase pathways (Dong et al., 2022). Ultimately leading to increased expression of inflammatory factors such as IL-1β, IL-2, IL-6, and TNF-α (Dong et al., 2022) and activation of astrocytes (Ott et al., 2014). Some drugs are considered to improve NF-κB activation caused by the above signaling pathways, reduce the activation of astrocytes, and inhibit the release of inflammatory factors, including Galantine (Liu et al., 2018), Juglanin (Zhou et al., 2016; Zhang and Xu, 2018), and Hesperetin (Muhammad et al., 2019; Evans et al., 2022), etc.

Moreover, recent research has found an association between increased expression of HMG20A [a chromatin factor that regulates genome expression by establishing active or silent chromatin (Mellado-Gil et al., 2018)] and the neuroprotective effect mediated by astrocyte activation. Although the specific reasons remain unclear, chronic hyperglycemia is believed to decrease the levels of HMG20A (Lorenzo et al., 2021), increasing neuronal susceptibility to stress-induced apoptosis. The lysine-specific demethylase 1 (a chromatin-modifying enzyme) inhibitor ORY1001 (Iadademstat) can mimic the role of HMG20A (Salamero et al., 2020; Noce et al., 2023), thereby reversing this imbalance.

Tight junctions of endothelial cells and blood-brain barrier (BBB): The BBB is a structural barrier located at the interface between brain tissue and blood, consisting of endothelial cells, basement membrane, pericytes, and astrocyte endfeet. The protective function of the BBB often leads to microvascular damage in diabetes preceding damage to brain neural tissue. Hyperglycemia can lead to BBB leakage by downregulating tight junction proteins between endothelial cells (Yoo et al., 2016), exposing various cell components of the NVU to a harmful environment. Additionally, this process further worsens BBB dysfunction by disrupting the adhesion relationship between the endothelial basement membrane and cell components (Gandhi et al., 2010; Chronopoulos et al., 2011) and increasing pericyte apoptosis (Shah et al., 2013; Price et al., 2017). Consequently, the brain tissue becomes more vulnerable to attack by peripheral immune cells, inflammatory factors, and ROS, ultimately leading to damage to the NVC (Bertram et al., 2016).

Previous studies have suggested that elevated blood glucose leads to BBB impairment by downregulating tight junction proteins between endothelial cells (Yoo et al., 2016). However, recent research has observed a significant increase in tight junction proteins, including occludin, on extracellular vesicles derived from endothelial cells isolated from the serum of T1DM mice (Rom et al., 2020). Additionally, the levels of occludin mRNA were markedly elevated in isolated micro-vessels (Rom et al., 2020). This indicates that high blood glucose may result in abnormal membrane distribution of tight junction proteins rather than a decrease in their expression. However, the specific mechanisms underlying this distribution abnormality remain unclear.

Gap junctions (GJs): GJs allow charged ions to pass freely, and GJs between vascular wall cells are a crucial structure for transmitting vasodilation signals. Due to the limited contractile function of capillaries, vascular dilation signals (such as K+-induced hyperpolarization currents and Ca2+ waves) transmitted through GJs to arterioles with stronger contractile function play a particularly important role in NVC. Kovacs-Oller et al. (2020) found that in the retinal capillaries of diabetic mice, the expression of GJs in pericytes was downregulated. This led to a limitation in GJ-dependent Ca2+ waves and vascular constriction responses (Kovacs-Oller et al., 2020), although the specific mechanism remains unclear. Interestingly, pericytes primarily connect with other neighboring pericytes and endothelial cells, with fewer connections to arterial SMCs. This exclusive connection reduces blood “stealing” from other branches (perfuse other areas but from the same arterioles) which ensures the spatial accuracy of NVC (Kovacs-Oller et al., 2020). Diabetes disrupts this accuracy, preventing the effective concentration of blood supply in regions with active neural function. Although this change in the retina has not been validated in cerebral NVC, imaging studies in humans with T2DM suggest a potential disruption in the spatial distribution of CBF (Tiehuis et al., 2008; Wang et al., 2021). This disruption implies that while overall cerebral perfusion may not decrease significantly, regional cerebral perfusion in various locations, including the occipital lobe and regions involving the default mode network, may be impaired.

Connexin43 (Cx43) is the most common type of gap junction protein in the human body, expressed in all types of vascular cells (Mugisho et al., 2017; Sedovy et al., 2023). Previous extensive research has shown that a high glucose environment damages gap junctional intercellular communication (GJIC) in both endothelial cells (Bobbie et al., 2010; Tien et al., 2013) and pericytes (Ivanova et al., 2017; Kovacs-Oller et al., 2020). This is associated with downregulation of Cx43 expression (Tien et al., 2013; Ivanova et al., 2017) and PKC-dependent overphosphorylation (Nimlamool et al., 2015; Shibata et al., 2019), which may promote proteasome-dependent degradation of Cx43 (Fernandes et al., 2004). Recently, Pan et al. (2022) found that high glucose downregulates endothelial cell Cx43 expression through activation of the RhoA/ROCK1/pMLC signaling pathway, and ROCK inhibitors significantly improve endothelial function. Additionally, Kim et al. (2020) reported that high glucose conditions increase the expression of Rab20 (a protein believed to regulate intracellular transport of Cx43) in retinal endothelial cells. Upregulation of Rab20 reduces the localization of Cx43 on the cell surface, thereby impairing GJIC (Kim et al., 2020). Homme et al. (2021) found that NF-κB inhibitors significantly reduce the degradation of retinal vascular Cx43 in T1DM mice. The Cx43 GJ decoupling inhibitor danegaptide improves GJIC in retinal vessels under high glucose conditions and reduces cell apoptosis (Kim et al., 2018). Furthermore, establishing an inducible specific ectopic Cx43 expression system in endothelial cells can compensate for the reduction of endogenous Cx43, providing a potentially powerful tool for treating diabetic microcirculatory defects (Ivanova et al., 2022). These studies collectively suggest that DM damages Cx43 and its mediated GJIC, providing further research directions for improving this imbalance. Although GJIC between vascular cells is significant for NVC (Alarcon-Martinez et al., 2020), more research is needed to validate the effects of these DM-related changes on NVC.

### 4.2 Impaired NVC signaling and calcium cascade in astrocytes

The generation of neuronal action potentials is the initiating factor for NVC. When neurons are activated by the excitatory neurotransmitter glutamate, the same signal also activates the metabolic glutamate receptor 5 (mGluR5) on neighboring astrocytes and leads to an increase in intracellular Ca2+ concentration through the classic inosine phosphate 4, 5-diphosphate (PIP2) -inositol triphosphate (IP3) -Ca2 + cascade [the source of calcium ions is still controversial (Bazargani and Attwell, 2016)]. Subsequently, the elevated intracellular Ca2+ concentration in the endfeet activates phospholipase D2, releasing arachidonic acid (AA). Subsequently, AA is converted to prostaglandins (e.g., PGE2, PGI2) and epoxyeicosatrienoic acids (EETs), which are vasodilatory substances acting on blood vessels through the cyclooxygenase and cytochrome P450 pathways. These pathways are considered crucial for astrocyte-mediated neurogenic capillary dilation (Biesecker et al., 2016; Rungta and Charpak, 2016). Since brain capillaries have a large surface area and are directly adjacent to brain tissue, they are considered the optimal site for implementing NVC (Iadecola, 2017). Therefore, vasodilation mediated by the above pathways is believed to be a critically important mechanism for NVC (Iadecola, 2017).

Elevated blood glucose levels have been shown to increase the expression of mGluR5 receptors in the cortical region of the adult rat brain (Joseph et al., 2008; Balakrishnan et al., 2009) while reducing the expression of mGluR5 in the striatum and hippocampus (Balakrishnan et al., 2009). However, this disruption of glutamate receptors may not affect NVC. The Ca2+ signaling in astrocytes is a controversial aspect of neuroscience (Bazargani and Attwell, 2016). In the cell bodies of astrocytes adjacent to neuronal synapses, activation of IPR2 results in the release of Ca2+ from the endoplasmic reticulum (Fiacco et al., 2007; Petravicz et al., 2008). Studies have shown that the knockout of the IPR2 gene does not impact NVC (Nizar et al., 2013; Bonder and McCarthy, 2014), and some researchers suggest that the slow rise in intracellular Ca2+ (Schummers et al., 2008; Schulz et al., 2012) may not generate rapid blood flow responses. These conclusions rule out the possibility that the [Ca2+] I wave generated by the release of stored Ca2+ propagates to the extremities of adjacent blood vessels. Nevertheless, rapid transient changes in Ca2+ have indeed been observed before vasodilation (Lind et al., 2013; Otsu et al., 2015), which may be partially dependent on ion channels on the cell membrane, such as transient receptor potential ankyrin 1 (TRPA1) channels, neurotransmitter-gated channels (e.g., NMDA) (Bazargani and Attwell, 2016), P2X receptors (Kisler et al., 2017) and(or) transient receptor potential vanilloid 4 (TRPV4) channels (Dunn et al., 2013; Diaz et al., 2019), mediating transient increases in [Ca2+] (Bazargani and Attwell, 2016). However, it remains unclear how diabetes affects these ion channels and foot process Ca2+ signaling. Subsequent research should focus on the causes of foot process Ca2+ signals and diabetes-related alterations. Additionally, the diabetes-related changes in the production of vasodilatory substances (PG and EETs) by astrocytes, which may diffuse to nearby vessels, leading to vasodilation, also warrant investigation.

### 4.3 Potassium ion signal and hyperpolarization current in vascular wall cells

Elevation of Ca2+ in astrocytic endfeet activates large-conductance calcium-activated potassium (BKCa) channels, leading to K+ efflux and an increase in extracellular K+ concentration in the space between the endfeet and vascular wall cells (Kisler et al., 2017). The elevated external K+ activates inward rectifying potassium channel 2.1 (Kir2.1) on vessel wall cells (endothelial cells, SMCs, and perivascular cells) (Longden and Nelson, 2015). The K+ efflux causes hyperpolarization and propagates hyperpolarization signals through gap junctions (Paulson and Newman, 1987; Longden et al., 2017; Figure 1). Simultaneously, it induces the closure of voltage-dependent Ca2+ channels on SMCs or perivascular cells, reducing Ca2+ influx and leading to localized vasodilation (Longden and Nelson, 2015).

Currently, there is no research indicating whether diabetes impairs the BKCa channels of astrocytes. However, studies have suggested a reduction in the functionality of Kir2.1 channels in cerebral arterioles of streptozotocin-induced T1DM rats (Mayhan et al., 2004), and this reduction is related to the increase of PKC activity caused by selective up-regulation of PKC-α (a subtype of PKC) (Vetri et al., 2013, 2017). Recent research has found reduced Kir2.1 expression in the cerebral microvasculature of Alzheimer’s disease (AD) rats, leading to early impairment of NVC in AD rats (Van Den Berg et al., 2023). Considering the common metabolic defects shared by AD and T2DM, such as impaired glucose metabolism, insulin resistance, and mitochondrial dysfunction (Carvalho and Moreira, 2023), further experiments are needed to determine whether T2DM similarly results in reduced Kir2.1 expression in the cerebral microvascular endothelium.

Vascular SMCs and pericytes also experience hyperpolarization and relaxation induced by the activation of BKCa channels by EETs from astrocytes (Kisler et al., 2017). In animal models of both type 1 diabetes mellitus (Liu, 2002; Fernández-Velasco et al., 2014) and T2DM (Wang et al., 2010; Nieves-Cintrón et al., 2017; Torabi et al., 2021), the activity of BKCa channels on cerebral vascular SMCs is reduced. This reduction may be associated with decreased expression of the β1 subunit of BKCa channels (Wang et al., 2010), increased ROS (Liu, 2002), and elevated PKC activity (Vetri et al., 2013, 2017). In general, diabetes-induced NVC damage may be related to the inhibition of BKCa and Kir2.1 channels caused by increased PKC activity (Vetri et al., 2012, 2017). Future targets may be diabetes-related changes in PKC subtypes and the specific pathways that lead to impaired NVC.

Additionally, adenosine, a metabolic product of neural activity, acts on A2A and A2B adenosine receptors in vascular SMCs, leading to vasodilation by activating ATP-sensitive potassium (KATP) channels (Ottolini et al., 2019), which may also contribute to NVC. Diabetes-related oxidative stress can lead to s-glutathionylation of the Kir6.1 subunit of KATP channels, inhibiting channel activity and impairing vasodilation in renal, hepatic, and cardiac arterioles (Yang et al., 2011; Li et al., 2015). Whether such alterations exist in cerebral vasculature and their impact on NVC requires further experimental validation.

From potassium signals to vasodilation: Lastly, K+ signaling relies on voltage-gated Ca2+ channels (VGCCs) on vascular SMCs\perivascular cells to achieve vasodilation (Longden and Nelson, 2015). Studies have indicated that T1DM may impair the function of VGCCs in afferent arterioles of the rat renal glomerulus (Carmines et al., 1996; Carmines and Fujiwara, 2002), while simultaneously leading to an upregulation in the expression and function of VGCC proteins in retinal ganglion cells (Wang Y.-C. et al., 2023). This seems to underscore the differential impact of diabetes on channel functionality across different tissues. However, there is currently a lack of research on the effects of diabetes on VGCCs in the vascular wall cells of the brain.

**FIGURE 2:**
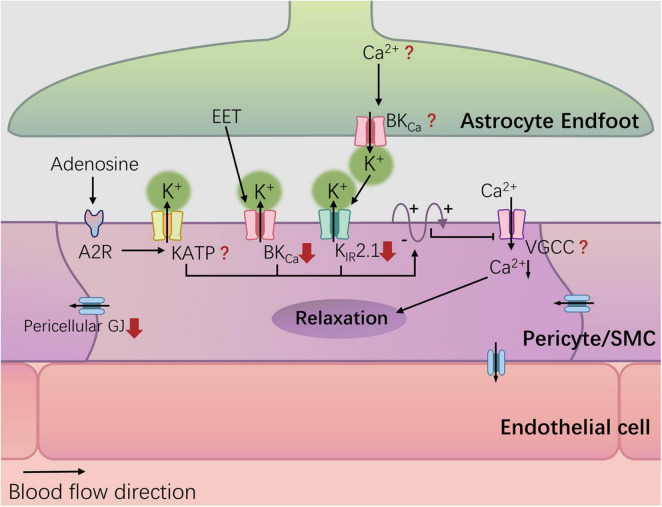
Potassium channel and diabetes-related changes. Hyperglycemia can damage NVC by damaging potassium ion channels in the blood vessel wall, including Kir2.1, and BKCa, and down-regulating the gap junction (GJ) protein of pericellular cells. The red arrows represent diabetes-related changes.

In summary, diabetes can damage NVC by impairing potassium ion channels on the vascular wall, including Kir2.1 (Vetri et al., 2017), BKCa (Wang et al., 2010; Torabi et al., 2021), and downregulating gap junction proteins in perivascular cells (Figure 2). Other crucial structures, such as BKCa channels on astrocyte end-feet, endothelial gap junctions, and VGCCs on vascular SMCs/perivascular cells, remain unknown in terms of diabetes-related changes. It is important to note that most studies have not validated the impact of corresponding alterations on NVC. The influence of a hyperglycemic environment on the nervous system and cerebral circulation is often systemic, and given that NVC involves concurrent signaling through known and unknown pathways, the contribution of a single signal loss to neurovascular decoupling is yet to be determined.

### 4.4 Impaired NO pathway

Excitatory neurotransmitter glutamate, through binding to N-methyl-D-aspartate (NMDA) receptors on the cell membrane increases intracellular calcium ion influx. Calcium-dependent enzymes such as nitric oxide synthase (NOS) are activated, leading to the synthesis of nitric oxide (NO) and inducing vasodilation (Iadecola, 2017; Figure 1). Since the role of NO in capillary dilation is still a matter of debate (Kisler et al., 2017), the neuronal NMDA receptor-mediated neuronal nitric oxide synthase (nNOS) pathway may primarily play a role in activity-induced arteriolar dilation. It has been found that hyperglycemia upregulates NMDA receptors in the hippocampus (Wang et al., 2019), but the effect of this change on NVC is still unknown.

In NVC, NO is not only produced through the neuronal nNOS pathway but also via the endothelial nitric oxide synthase (eNOS) pathway. ATP belongs to the purinergic receptor (P2Y) agonists in endothelial cells. When ATP is released from astrocytes in response to neuronal activation, it can trigger the production of NO in endothelial cells, leading to vasodilation (Toth et al., 2015a,b; Wells et al., 2015). Different vessels have specific physiological roles, and there are significant variations in purinergic regulatory mechanisms among different vessels (Burnstock and Ralevic, 2014). Currently, research on the impact of T2DM on P2Y receptors and NO generation in cerebral vascular endothelium is relatively limited.

A study employed NO microelectrodes and laser Doppler probes to simultaneously measure changes in NO and CBF in the hippocampus of Goto-Kakizaki(GK) rats (a diabetic rat model), revealing a reduction in the increase of NO following glutamate activation and impairment of NVC (Gonçalves et al., 2022). Another investigation utilized laser Doppler flowmetry to measure CBF in the somatosensory cortex of GK rats after whisker stimulation demonstrating that NVC impairment was accompanied by elevated reactive oxygen and nitrogen species (RONS), nitrotyrosine, and peroxynitrite (a product of the reaction between oxygen free radicals and NO) in both plasma and cerebral arteries (Kelly-Cobbs et al., 2012). Similar NVC impairment was observed in the somatosensory cortex of healthy mice treated with NOS inhibitors (Tarantini et al., 2015), while supplementation of NO was found to alleviate NVC damage induced by prolonged hyperglycemia in zebrafish (Chhabria et al., 2020). These findings suggest that the impairment of NVC may stem from reduced bioavailability and/or synthesis of NO due to oxygen-free radicals.

Endothelial nitric oxide synthase (eNOS) uncoupling and NVC impairment: The reduced synthesis of NO in diabetic patients is associated with a deficiency in tetrahydrobiopterin (BH4) (Wu and Meininger, 2009). BH4 serves as a cofactor for three isoforms of NOS (eNOS, nNOS, and inducible NOS), and its deficiency can lead to NOS uncoupling (Bendall et al., 2014). NOS uncoupling triggers the production of superoxide anions (O2-) through a NADPH oxidase-like system, further exacerbating oxidative stress (Guerby et al., 2021; Janaszak-Jasiecka et al., 2023). The deficiency of BH4 (imbalanced BH4/BH2 ratio) (Kuzkaya et al., 2003; Fanet et al., 2021), and the consequent reduction in NO bioavailability, create a vicious cycle of oxidative stress. Regarding nNOS, research has found that high glucose levels lead to a reduction in the expression of guanosine triphosphate cyclohydrolase-1 (GCH-1, the synthetic enzyme for BH4) and nNOS by inhibiting the enteric neuron PI3K/AKT/nuclear factor erythroid 2-related factor 2 (Nrf2) signaling pathway (Sampath et al., 2022). The natural activator of Nrf2, cinnamaldehyde, can reverse this damage, restoring enteric neuron BH4/nNOS functionality (Sampath et al., 2019, 2022). Additionally, Butein can elevate Nrf2 by activating the PI3K/Akt pathway, mediating protective effects on hippocampal neurons (Lee and Jeong, 2016). For eNOS, Resveratrol can also reverse endothelial eNOS uncoupling by activating the aforementioned signaling pathway (Wallerath et al., 2002; Parsamanesh et al., 2021). In general, improving Nrf2 may have broad applicability for preventing NOS uncoupling in neurons (Xiong et al., 2015; Lee and Jeong, 2016) and endothelial cells (Gao and Mann, 2009; Zhang Q. et al., 2021). However, the specific role of nNOS and/or eNOS uncoupling resulting from BH4 deficiency in NVC remains unknown. Subsequent studies could investigate this by examining the impact of drugs that improve BH4/NOS on brain NVC, providing reverse validation of the relationship between BH4/NOS impairment and NVC.

## 5 Hypoglycemia and impaired NVC

Early fMRI studies on healthy humans (Anderson et al., 2006; Driesen et al., 2007) and rats (Kennan et al., 2000) found a reduced blood flow response to stimulation during low blood sugar, attributing this phenomenon to impaired NVC. However, during hypoglycemia, the basal CBF increases to ensure glucose supply to brain neurons (McManus et al., 2020; Nippert et al., 2022), thought to be related to adenosine-induced increases in astrocyte Ca2+ signaling (Nippert et al., 2022). The mentioned studies did not record neuronal activity, and the observed changes in blood flow responses by fMRI might also be influenced by the increased basal CBF during hypoglycemia (McManus et al., 2020; Nippert et al., 2023). In a recent study, Nippert et al. (2023) simultaneously monitored the responses of neurons and blood vessels in the somatosensory cortex of awake healthy mice to whisker stimulation. They concluded that neuronal activity and NVC remain unchanged during hypoglycemia (Nippert et al., 2023). Although more experiments are needed to confirm this, Nippert et al.’s conclusion may be one of the more convincing for now.

The energy synthesis of astrocytes themselves is crucial for supporting NVC and neurons. On one hand, ATP and its metabolites serve as important signaling molecules for intercellular communication among astrocytes and NVC (Robinson and Jackson, 2016). On the other hand, during periods of high neuronal activity, astrocytes can replenish the neurotransmitter pool in neurons (Bélanger et al., 2011) and supply energy substrates to axons (González-Gutiérrez et al., 2020; Guo et al., 2021). Recurrent low glucose (RLG) reduces the expression of Sirtuin-3 (SIRT3) (a key deacetylase for mitochondrial proteins) in astrocytes cultured in a hyperglycemic environment, impairing mitochondrial homeostasis (Zhou et al., 2018; Gao et al., 2022), leading to disturbances in neuronal nutrition and neuronal cell death (Gao et al., 2021). Overexpression of SIRT3 can improve the function of astrocytic mitochondria by increasing mitochondrial bioenergetic status and reducing mitochondrial oxidative stress levels (Gao et al., 2022). Recently, some 1,4-dihydropyridine-based compounds have been suggested as specific activators of SIRT3 (Suenkel et al., 2022; Zwergel et al., 2023), providing a framework for drug research to treat RLG-induced neuronal injury and NVC impairment.

## 6 Treatment

Antidiabetic medications, such as metformin (Secnik et al., 2021), sodium-dependent glucose co-transporter 2 inhibitors (SGLT2i) (Rizzo et al., 2022), dipeptidyl peptidase-4 inhibitors (DPP-4 inhibitors) (Ma et al., 2015), and glucagon-like peptide-1 receptor agonists (GLP-1RAs) (Cukierman-Yaffe et al., 2020), are believed to have potential benefits in improving cognitive function. However, it remains unclear whether the improvement in NVC is one of the mechanisms. Some drugs have been found to enhance NVC, such as the SGLT2 inhibitor tofogliflozin (Hanaguri et al., 2022c), peroxisome proliferator-activated receptor alpha (PPARα) agonist Fenofibrate (Hanaguri et al., 2022a), and Lutein (Hanaguri et al., 2022b), which improve retinal NVC in Type 2 Diabetic Mice. Resveratrol, a polyphenolic compound primarily found in fruits, can enhance NVC and cognitive function in T2DM (Wong et al., 2016; Tu et al., 2023). This is not only related to the improvement of endothelial BH4/eNOS mentioned above, but also to the inhibition of the activation of NF-κB signaling to play an anti-inflammatory role (Parsamanesh et al., 2021). Nicotinamide mononucleotide (Kiss et al., 2020a) and dipeptide (Rom et al., 2018) are considered to improve NVC by enhancing endothelial function. Modulating the NO pathway in the human body may be an effective approach for treating NVC disruptions associated with T2DM (Lourenço and Laranjinha, 2021). Nitroprusside can ameliorate the adverse effects of persistent hyperglycemia on NVC in zebrafish (Chhabria et al., 2020). In addition to providing NO directly, nitroprusside has also been found to reduce astrocyte reactivity (Chhabria et al., 2020). Most of these drugs are still in the experimental stage. primarily in animal studies, and further research, including clinical trials, is needed for validation. Additionally, the development of mitochondria-targeted antioxidants can enhance mitochondrial antioxidant defenses, potentially increasing the bioavailability of NO and rescuing NVC responses in aged mice (Csiszar et al., 2019). Developing antioxidants targeted at mitochondria could be a promising direction for future drug development.

## 7 Conclusion

**FIGURE 3:**
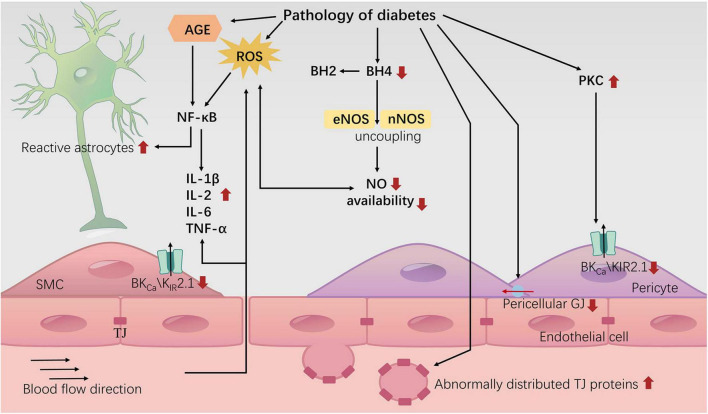
Neurovascular uncoupling in diabetes. The diagram represents the current knowledge of neurovascular decoupling associated with diabetes. TJ: tight junction; GJ: gap junction.

In summary, the diabetic environment may impair NVU structure and NVC signal transduction, leading to cognitive decline (Figure 3). Research on therapeutic drugs targeting known mechanisms is still in the experimental stage. The intricate network of interacting factors contributing to NVC damage induced by diabetes requires further in-depth investigation. In addition to NVC, we also need to consider a relatively new model known as vascular-neuronal coupling (VNC), where changes in vascular tension can influence neuronal electrical activity (Kim et al., 2016). Diabetes is widely recognized as a risk factor for brain arteriolosclerosis (B-ASC) (Borshchev et al., 2019). B-ASC is characterized by pathological thickening of the small arterial walls and decreased compliance, and it is associated with cognitive impairment (Blevins et al., 2021). Could the reduction in vascular compliance caused by B-ASC lead to cognitive decline through VNC? This is a relatively unexplored area that may offer new insights into the mechanisms of diabetes-related cognitive impairment.

## Author contributions

LF: Writing – original draft, Writing – review & editing. LG: Resources, Supervision, Writing – original draft, Writing – review & editing.

## References

[B1] Alarcon-Martinez L. Villafranca-Baughman D. Quintero H. Kacerovsky J. Dotigny F. Murai K. (2020). Interpericyte tunnelling nanotubes regulate neurovascular coupling. *Nature* 585 91–95. 32788726 10.1038/s41586-020-2589-x

[B2] Anderson A. W. Heptulla R. Driesen N. Flanagan D. Goldberg P. Jones T. (2006). Effects of hypoglycemia on human brain activation measured with fMRI. *Magn. Reson. Imaging* 24 693–697. 16824963 10.1016/j.mri.2006.03.013

[B3] Balakrishnan S. Kumar T. P. Paulose C. (2009). Glutamate (mGluR-5) gene expression in brain regions of streptozotocin induced diabetic rats as a function of age: Role in regulation of calcium release from the pancreatic islets in vitro. *J. Biomed. Sci.* 16:99. 10.1186/1423-0127-16-99 19903331 PMC2779807

[B4] Balasubramanian P. Kiss T. Tarantini S. Nyúl-Tóth Á Ahire C. Yabluchanskiy A. (2021). Obesity-induced cognitive impairment in older adults: A microvascular perspective. *Am. J. Physiol. Heart Circ. Physiol.* 320 H740–H761. 10.1152/ajpheart.00736.2020 33337961 PMC8091942

[B5] Bangen K. J. Werhane M. Weigand A. Edmonds E. Delano-Wood L. Thomas K. (2018). Reduced regional cerebral blood flow relates to poorer cognition in older adults with type 2 diabetes. *Front. Aging Neurosci.* 10:270. 10.3389/fnagi.2018.00270 30250430 PMC6139361

[B6] Bazargani N. Attwell D. (2016). Astrocyte calcium signaling: The third wave. *Nat. Neurosci.* 19 182–189. 26814587 10.1038/nn.4201

[B7] Bekar L. K. Wei H. S. Nedergaard M. (2012). The locus coeruleus-norepinephrine network optimizes coupling of cerebral blood volume with oxygen demand. *J. Cereb. Blood Flow Metab.* 32 2135–2145. 10.1038/jcbfm.2012.115 22872230 PMC3519408

[B8] Bélanger M. Allaman I. Magistretti P. J. (2011). Brain energy metabolism: Focus on astrocyte-neuron metabolic cooperation. *Cell Metab.* 14 724–738. 22152301 10.1016/j.cmet.2011.08.016

[B9] Bendall J. K. Douglas G. McNeill E. Channon K. M. Crabtree M. J. (2014). Tetrahydrobiopterin in cardiovascular health and disease. *Antioxid. Redox Signal.* 20 3040–3077. 24294830 10.1089/ars.2013.5566 PMC4038990

[B10] Bertram S. Brixius K. Brinkmann C. (2016). Exercise for the diabetic brain: How physical training may help prevent dementia and Alzheimer’s disease in T2DM patients. *Endocrine* 53 350–363. 10.1007/s12020-016-0976-8 27160819

[B11] Biesecker K. R. Srienc A. Shimoda A. Agarwal A. Bergles D. Kofuji P. (2016). Glial cell calcium signaling mediates capillary regulation of blood flow in the retina. *J. Neurosci.* 36 9435–9445. 10.1523/JNEUROSCI.1782-16.2016 27605617 PMC5013190

[B12] Biessels G. J. Despa F. (2018). Cognitive decline and dementia in diabetes mellitus: Mechanisms and clinical implications. *Nat. Rev. Endocrinol.* 14 591–604. 30022099 10.1038/s41574-018-0048-7 PMC6397437

[B13] Blevins B. L. Vinters H. Love S. Wilcock D. Grinberg L. Schneider J. (2021). Brain arteriolosclerosis. *Acta Neuropathol.* 141 1–24. 33098484 10.1007/s00401-020-02235-6 PMC8503820

[B14] Bobbie M. W. Roy S. Trudeau K. Munger S. Simon A. Roy S. (2010). Reduced connexin 43 expression and its effect on the development of vascular lesions in retinas of diabetic mice. *Investig. Opthalmol. Vis. Sci.* 51:3758. 10.1167/iovs.09-4489 20130277 PMC2904019

[B15] Bonder D. E. McCarthy K. D. (2014). Astrocytic Gq-GPCR-linked IP 3 R-dependent Ca 2+ signaling does not mediate neurovascular coupling in mouse visual cortex in vivo. *J. Neurosci.* 34 13139–13150. 10.1523/JNEUROSCI.2591-14.2014 25253859 PMC4172806

[B16] Borshchev Y. Uspensky Y. P. Galagudza M. M. (2019). Pathogenetic pathways of cognitive dysfunction and dementia in metabolic syndrome. *Life Sci.* 237:116932. 10.1016/j.lfs.2019.116932 31606384

[B17] Brundel M. van den Berg E. Reijmer Y. de Bresser J. Kappelle L. Biessels G. (2012). Cerebral haemodynamics, cognition and brain volumes in patients with type 2 diabetes. *J. Diabetes Complications* 26 205–209. 22520398 10.1016/j.jdiacomp.2012.03.021

[B18] Burillo J. Fernández-Rhodes M. Piquero M. López-Alvarado P. Menéndez J. Jiménez B. (2021). Human amylin aggregates release within exosomes as a protective mechanism in pancreatic β cells: Pancreatic β-hippocampal cell communication. *Biochim. Biophys. Acta BBA Mol. Cell Res.* 1868:118971. 10.1016/j.bbamcr.2021.118971 33515645

[B19] Burnstock G. Ralevic V. (2014). Purinergic signaling and blood vessels in health and disease. *Pharmacol. Rev.* 66 102–192. 24335194 10.1124/pr.113.008029

[B20] Canna A. Esposito F. Tedeschi G. Trojsi F. Passaniti C. di Meo I. (2022). Neurovascular coupling in patients with type 2 diabetes mellitus. *Front. Aging Neurosci.* 14:976340. 10.3389/fnagi.2022.976340 36118711 PMC9476313

[B21] Carmines P. K. Fujiwara K. (2002). Altered electromechanical coupling in the renal microvasculature during the early stage of diabetes mellitus. *Clin. Exp. Pharmacol. Physiol.* 29 143–148. 11906474 10.1046/j.1440-1681.2002.03616.x PMC2570963

[B22] Carmines P. K. Ohishi K. Ikenaga H. (1996). Functional impairment of renal afferent arteriolar voltage-gated calcium channels in rats with diabetes mellitus. *J. Clin. Invest.* 98 2564–2571. 10.1172/JCI119075 8958219 PMC507714

[B23] Carvalho C. Moreira P. I. (2023). Metabolic defects shared by Alzheimer’s disease and diabetes: A focus on mitochondria. *Curr. Opin. Neurobiol.* 79:102694. 10.1016/j.conb.2023.102694 36842275

[B24] Castaneda-Vega S. Beer-Hammer S. Leiss V. Napieczyñska H. Vuozzo M. Schmid A. (2023). Cerebrovascular Gi proteins protect against brain hypoperfusion and collateral failure in cerebral ischemia. *Mol. Imaging Biol.* 25 363–374. 10.1007/s11307-022-01764-8 36074223 PMC10006265

[B25] Chen H. Wan Y. Zhou S. Lu Y. Zhang Z. Zhang R. (2012). Endothelium-specific SIRT1 overexpression inhibits hyperglycemia-induced upregulation of vascular cell senescence. *Sci. China Life Sci.* 55 467–473. 10.1007/s11427-012-4329-4 22744176

[B26] Chhabria K. Plant K. Bandmann O. Wilkinson R. Martin C. Kugler E. (2020). The effect of hyperglycemia on neurovascular coupling and cerebrovascular patterning in zebrafish. *J. Cereb. Blood Flow Metab.* 40 298–313. 10.1177/0271678X18810615 30398083 PMC6985997

[B27] Chronopoulos A. Trudeau K. Roy S. Huang H. Vinores S. Roy S. (2011). High glucose-induced altered basement membrane composition and structure increases trans-endothelial permeability: Implications for diabetic retinopathy. *Curr. Eye Res.* 36 747–753. 10.3109/02713683.2011.585735 21780924

[B28] Csiszar A. Yabluchanskiy A. Ungvari A. Ungvari Z. Tarantini S. (2019). Overexpression of catalase targeted to mitochondria improves neurovascular coupling responses in aged mice. *GeroScience* 41 609–617. 10.1007/s11357-019-00111-0 31643012 PMC6885076

[B29] Cui Y. Li Q. Yamada H. Watanabe Y. Kataoka Y. (2013). Chronic degeneration of dorsal raphe serotonergic neurons modulates cortical spreading depression: A possible pathophysiology of migraine. *J. Neurosci. Res.* 91 737–744. 10.1002/jnr.23209 23456883

[B30] Cui Y. Liang X. Gu H. Hu Y. Zhao Z. Yang X. (2017). Cerebral perfusion alterations in type 2 diabetes and its relation to insulin resistance and cognitive dysfunction. *Brain Imaging Behav.* 11 1248–1257. 27714551 10.1007/s11682-016-9583-9 PMC5653700

[B31] Cukierman-Yaffe T. Gerstein H. Colhoun H. Diaz R. García-Pérez L. Lakshmanan M. (2020). Effect of dulaglutide on cognitive impairment in type 2 diabetes: An exploratory analysis of the REWIND trial. *Lancet Neurol.* 19 582–590. 10.1016/S1474-4422(20)30173-3 32562683

[B32] Dao L. Choi S. Freeby M. (2023). Type 2 diabetes mellitus and cognitive function: Understanding the connections. *Curr. Opin. Endocrinol. Diabetes Obes.* 30 7–13. 36385094 10.1097/MED.0000000000000783

[B33] Despa F. Goldstein L. B. (2021). Amylin dyshomeostasis hypothesis: Small vessel–type ischemic stroke in the setting of type-2 diabetes. *Stroke* 52 e244–e249. 10.1161/STROKEAHA.121.034363 33947210 PMC8154741

[B34] Dewenter A. Jacob M. Cai M. Gesierich B. Hager P. Kopczak A. (2023). Disentangling the effects of Alzheimer’s and small vessel disease on white matter fibre tracts. *Brain* 146 678–689. 10.1093/brain/awac265 35859352 PMC9924910

[B35] Diaz J. R. Kim K. J. Brands M. W. Filosa J. A. (2019). Augmented astrocyte microdomain Ca 2+ dynamics and parenchymal arteriole tone in angiotensin II-infused hypertensive mice. *Glia* 67 551–565. 10.1002/glia.23564 30506941 PMC7033753

[B36] Dong H. Zhang Y. Huang Y. Deng H. (2022). Pathophysiology of RAGE in inflammatory diseases. *Front. Immunol.* 13:931473. 10.3389/fimmu.2022.931473 35967420 PMC9373849

[B37] Dorner G. T. Garhöfer G. Huemer K. Riva C. Wolzt M. Schmetterer L. (2003). Hyperglycemia affects flicker-induced vasodilation in the retina of healthy subjects. *Vision Res.* 43 1495–1500. 10.1016/s0042-6989(03)00170-6 12767316

[B38] Driesen N. R. Goldberg P. Anderson A. Tang L. Flanagan D. Sherwin R. (2007). Hypoglycemia reduces the blood-oxygenation level dependent signal in primary auditory and visual cortex: A functional magnetic resonance imaging study. *J. Neurosci. Res.* 85 575–582. 10.1002/jnr.21146 17154420

[B39] Duarte J. V. Guerra C. Moreno C. Gomes L. Castelo-Branco M. (2023). Changes in hemodynamic response function components reveal specific changes in neurovascular coupling in type 2 diabetes. *Front. Physiol.* 13:1101470. 10.3389/fphys.2022.1101470 36703928 PMC9872943

[B40] Duarte J. V. Pereira J. Quendera B. Raimundo M. Moreno C. Gomes L. (2015). Early disrupted neurovascular coupling and changed event level hemodynamic response function in type 2 diabetes: An fMRI study. *J. Cereb. Blood Flow Metab.* 35 1671–1680. 10.1038/jcbfm.2015.106 26058698 PMC4640307

[B41] Dunn K. M. Hill-Eubanks D. C. Liedtke W. B. Nelson M. T. (2013). TRPV4 channels stimulate Ca 2 + -induced Ca 2+ release in astrocytic endfeet and amplify neurovascular coupling responses. *Proc. Natl. Acad. Sci. U.S.A.* 110 6157–6162. 10.1073/pnas.1216514110 23530219 PMC3625327

[B42] Evans J. A. Mendonca P. Soliman K. F. A. (2022). Neuroprotective effects and therapeutic potential of the citrus flavonoid hesperetin in neurodegenerative diseases. *Nutrients* 14:2228. 10.3390/nu14112228 35684025 PMC9183194

[B43] Fanet H. Capuron L. Castanon N. Calon F. Vancassel S. (2021). Tetrahydrobioterin (BH4) pathway: From metabolism to neuropsychiatry. *Curr. Neuropharmacol.* 19 591–609. 10.2174/1570159X18666200729103529 32744952 PMC8573752

[B44] Fernandes R. Girão H. Pereira P. (2004). High glucose down-regulates intercellular communication in retinal endothelial cells by enhancing degradation of connexin 43 by a proteasome-dependent mechanism. *J. Biol. Chem.* 279 27219–27224. 10.1074/jbc.M400446200 15123628

[B45] Fernández-Velasco M. Ruiz-Hurtado G. Gómez A. M. Rueda A. (2014). Ca2+ handling alterations and vascular dysfunction in diabetes. *Cell Calcium* 56 397–407. 25218935 10.1016/j.ceca.2014.08.007

[B46] Fiacco T. A. Agulhon C. Taves S. Petravicz J. Casper K. Dong X. (2007). Selective stimulation of astrocyte calcium in situ does not affect neuronal excitatory synaptic activity. *Neuron* 54 611–626. 17521573 10.1016/j.neuron.2007.04.032

[B47] Fletcher E. Gavett B. Harvey D. Farias S. Olichney J. Beckett L. (2018). Brain volume change and cognitive trajectories in aging. *Neuropsychology* 32 436–449. 29494196 10.1037/neu0000447 PMC6525569

[B48] Gandhi G. K. Ball K. K. Cruz N. F. Dienel G. A. (2010). Hyperglycaemia and diabetes impair gap junctional communication among astrocytes. *ASN Neuro* 2:AN20090048. 10.1042/AN20090048 20396375 PMC2839462

[B49] Gao L. Mann G. E. (2009). Vascular NAD(P)H oxidase activation in diabetes: A double-edged sword in redox signalling. *Cardiovasc. Res.* 82 9–20. 10.1093/cvr/cvp031 19179352

[B50] Gao R. Chen Z. Wu Y. Chen R. Zheng W. Qi L. (2022). SIRT3 alleviates mitochondrial dysfunction induced by recurrent low glucose and improves the supportive function of astrocytes to neurons. *Free Radic. Biol. Med.* 193 405–420. 10.1016/j.freeradbiomed.2022.10.313 36306990

[B51] Gao R. Ren L. Zhou Y. Wang L. Xie Y. Zhang M. (2021). Recurrent non-severe hypoglycemia aggravates cognitive decline in diabetes and induces mitochondrial dysfunction in cultured astrocytes. *Mol. Cell. Endocrinol.* 526:111192. 10.1016/j.mce.2021.111192 33545179

[B52] Gaspar J. M. Castilho Á Baptista F. I. Liberal J. Ambrósio A. F. (2010). Long-term exposure to high glucose induces changes in the content and distribution of some exocytotic proteins in cultured hippocampal neurons. *Neuroscience* 171 981–992. 10.1016/j.neuroscience.2010.10.019 20950673

[B53] Georgakis M. K. Harshfield E. Malik R. Franceschini N. Langenberg C. Wareham N. (2021). Diabetes mellitus, glycemic traits, and cerebrovascular disease: A Mendelian randomization study. *Neurology* 96 e1732–e1742. 33495378 10.1212/WNL.0000000000011555 PMC8055310

[B54] Gonçalves J. S. Seiça R. M. Laranjinha J. Lourenço C. F. (2022). Impairment of neurovascular coupling in the hippocampus due to decreased nitric oxide bioavailability supports early cognitive dysfunction in type 2 diabetic rats. *Free Radic. Biol. Med.* 193 669–675. 10.1016/j.freeradbiomed.2022.11.009 36372286

[B55] González-Gutiérrez A. Ibacache A. Esparza A. Barros L. F. Sierralta J. (2020). Neuronal lactate levels depend on glia-derived lactate during high brain activity in *Drosophila*. *Glia* 68 1213–1227. 10.1002/glia.23772 31876077

[B56] Gorelick P. B. Furie K. Iadecola C. Smith E. Waddy S. Lloyd-Jones D. (2017). Defining optimal brain health in adults: A presidential advisory from the American heart association/American stroke association. *Stroke* 48 e284–e303. 10.1161/STR.0000000000000148 28883125 PMC5654545

[B57] Guerby P. Tasta O. Swiader A. Pont F. Bujold E. Parant O. (2021). Role of oxidative stress in the dysfunction of the placental endothelial nitric oxide synthase in preeclampsia. *Redox Biol.* 40:101861. 10.1016/j.redox.2021.101861 PMC7873691 33548859

[B58] Guo H. Fan Z. Wang S. Ma L. Wang J. Yu D. (2021). Astrocytic A1/A2 paradigm participates in glycogen mobilization mediated neuroprotection on reperfusion injury after ischemic stroke. *J. Neuroinflammation* 18:230. 10.1186/s12974-021-02284-y 34645472 PMC8513339

[B59] Hamel E. (2006). Perivascular nerves and the regulation of cerebrovascular tone. *J. Appl. Physiol.* 100 1059–1064. 16467392 10.1152/japplphysiol.00954.2005

[B60] Han C. Lu Y. Wei Y. Wu B. Liu Y. He R. (2014). D-ribosylation induces cognitive impairment through RAGE-dependent astrocytic inflammation. *Cell Death Dis.* 5 e1117–e1117. 10.1038/cddis.2014.89 24625976 PMC3973213

[B61] Hanaguri J. Yokota H. Kushiyama A. Kushiyama S. Watanabe M. Yamagami S. (2022c). The effect of sodium-dependent glucose cotransporter 2 inhibitor tofogliflozin on neurovascular coupling in the retina in type 2 diabetic mice. *Int. J. Mol. Sci.* 23:1362. 10.3390/ijms23031362 35163285 PMC8835894

[B62] Hanaguri J. Nagai N. Yokota H. Kushiyama A. Watanabe M. Yamagami S. (2022a). Fenofibrate nano-eyedrops ameliorate retinal blood flow dysregulation and neurovascular coupling in type 2 diabetic mice. *Pharmaceutics* 14:384. 10.3390/pharmaceutics14020384 35214116 PMC8876509

[B63] Hanaguri J. Yokota H. Kushiyama A. Kushiyama S. Watanabe M. Yamagami S. (2022b). Beneficial effect of long-term administration of supplement with trapa bispinosa roxb. and lutein on retinal neurovascular coupling in type 2 diabetic mice. *Front. Physiol.* 13:788034. 10.3389/fphys.2022.788034 35283788 PMC8908418

[B64] Hayden M. (2019). Type 2 diabetes mellitus increases the risk of late-onset Alzheimer’s disease: Ultrastructural remodeling of the neurovascular unit and diabetic Gliopathy. *Brain Sci.* 9:262. 10.3390/brainsci9100262 31569571 PMC6826500

[B65] Hirabayashi N. Hata J. Ohara T. Mukai N. Nagata M. Shibata M. (2016). Association between diabetes and hippocampal atrophy in elderly Japanese: The Hisayama study. *Diabetes Care* 39 1543–1549. 27385328 10.2337/dc15-2800

[B66] Ho N. Brookshire B. R. Clark J. E. Lucki I. (2015). Indomethacin reverses decreased hippocampal cell proliferation in streptozotocin-induced diabetic mice. *Metab. Brain Dis.* 30 555–562. 25160865 10.1007/s11011-014-9611-7 PMC4344936

[B67] Homme R. P. Sandhu H. S. George A. K. Tyagi S. C. Singh M. (2021). Sustained inhibition of NF-κB activity mitigates retinal vasculopathy in diabetes. *Am. J. Pathol.* 191 947–964. 33640319 10.1016/j.ajpath.2021.01.016 PMC8101051

[B68] Howarth C. Mishra A. Hall C. N. (2021). More than just summed neuronal activity: How multiple cell types shape the BOLD response. *Philos. Trans. R. Soc. B Biol. Sci.* 376:20190630. 10.1098/rstb.2019.0630 33190598 PMC7116385

[B69] Hu B. Yan L. Sun Q. Yu Y. Zhang J. Dai Y. (2019). Disturbed neurovascular coupling in type 2 diabetes mellitus patients: Evidence from a comprehensive fMRI analysis. *Neuroimage Clin.* 22:101802. 10.1016/j.nicl.2019.101802 30991623 PMC6447740

[B70] Huang L. Zhang Q. Tang T. Yang M. Chen C. Tao J. (2021). Abnormalities of brain white matter in type 2 diabetes mellitus: A meta-analysis of diffusion tensor imaging. *Front. Aging Neurosci.* 13:693890. 10.3389/fnagi.2021.693890 34421572 PMC8378805

[B71] Huang Y. Liao Z. Lin X. Wu X. Chen X. Bai X. (2019). Overexpression of miR-146a might regulate polarization transitions of BV-2 cells induced by high glucose and glucose fluctuations. *Front. Endocrinol.* 10:719. 10.3389/fendo.2019.00719 31695681 PMC6817609

[B72] Hwang H. J. Kim N. Herman A. B. Gorospe M. Lee J.-S. (2022). Factors and pathways modulating endothelial cell senescence in vascular aging. *Int. J. Mol. Sci.* 23:10135. 10.3390/ijms231710135 PMC9456027 36077539

[B73] Hwang I. K. Kim I. Joo E. Shin J. Choi J. Won M. (2010). Metformin normalizes type 2 diabetes-induced decrease in cell proliferation and neuroblast differentiation in the rat dentate gyrus. *Neurochem. Res.* 35 645–650. 10.1007/s11064-009-0115-5 20069360

[B74] Hwang I. K. Yi S. Kim Y. Kim I. Lee I. Yoon Y. (2008). Reduced hippocampal cell differentiation in the subgranular zone of the dentate gyrus in a rat model of type ii diabetes. *Neurochem. Res.* 33 394–400. 10.1007/s11064-007-9440-8 17712629

[B75] Iadecola C. (2017). The neurovascular unit coming of age: A journey through neurovascular coupling in health and disease. *Neuron* 96 17–42. 10.1016/j.neuron.2017.07.030 28957666 PMC5657612

[B76] Ivanova E. Corona C. Eleftheriou C. Stout R. Jr. Körbelin J. Sagdullaev B. T. A. A. V. (2022). BR1 targets endothelial cells in the retina to reveal their morphological diversity and to deliver Cx43. *J. Comp. Neurol.* 530 1302–1317. 10.1002/cne.25277 34811744 PMC8969189

[B77] Ivanova E. Kovacs-Oller T. Sagdullaev B. T. (2017). Vascular pericyte impairment and connexin43 gap junction deficit contribute to vasomotor decline in diabetic retinopathy. *J. Neurosci.* 37 7580–7594. 10.1523/JNEUROSCI.0187-17.2017 28674171 PMC5551058

[B78] Janaszak-Jasiecka A. Płoska A. Wieroñska J. M. Dobrucki L. W. Kalinowski L. (2023). Endothelial dysfunction due to eNOS uncoupling: Molecular mechanisms as potential therapeutic targets. *Cell. Mol. Biol. Lett.* 28:21. 10.1186/s11658-023-00423-2 PMC9996905 36890458

[B79] Jeong H. W. Lee C. H. Kim D. H. (2022). Effect of white matter hyperintensities on daily function via depressive symptoms: A longitudinal study in patients with dementia including Alzheimer’s disease and subcortical ischemic vascular dementia. *Psychiatry Investig.* 19 687–694. 10.30773/pi.2022.0118 36059058 PMC9441457

[B80] Jha J. C. Ho F. Dan C. Jandeleit-Dahm K. (2018). A causal link between oxidative stress and inflammation in cardiovascular and renal complications of diabetes. *Clin. Sci.* 132 1811–1836. 10.1042/CS20171459 30166499

[B81] Jing J. Zhou Y. Pan Y. Cai X. Zhu W. Zhang Z. (2022). Reduced white matter microstructural integrity in prediabetes and diabetes: A population-based study. *eBioMedicine* 82:104144. 10.1016/j.ebiom.2022.104144 35810560 PMC9278067

[B82] Joseph A. Antony S. Paulose C. S. (2008). Increased glutamate receptor gene expression in the cerebral cortex of insulin induced hypoglycemic and streptozotocin-induced diabetic rats. *Neuroscience* 156 298–304. 18761060 10.1016/j.neuroscience.2008.07.022

[B83] Kelly-Cobbs A. I. Prakash R. Coucha M. Knight R. Li W. Ogbi S. (2012). Cerebral myogenic reactivity and blood flow in type 2 diabetic rats: Role of peroxynitrite in hypoxia-mediated loss of myogenic tone. *J. Pharmacol. Exp. Ther.* 342 407–415. 10.1124/jpet.111.191296 22570365 PMC3400801

[B84] Kennan R. P. Jacob R. J. Sherwin R. S. Gore J. C. (2000). Effects of hypoglycemia on functional magnetic resonance imaging response to median nerve stimulation in the rat brain. *J. Cereb. Blood Flow Metab.* 20 1352–1359. 10.1097/00004647-200009000-00010 10994857

[B85] Kim D. Lewis C. S. Sarthy V. P. Roy S. (2020). High-glucose-induced rab20 upregulation disrupts gap junction intercellular communication and promotes apoptosis in retinal endothelial and Müller cells: Implications for diabetic retinopathy. *J. Clin. Med.* 9:3710. 10.3390/jcm9113710 33227912 PMC7699280

[B86] Kim D. Mouritzen U. Larsen B. D. Roy S. (2018). Inhibition of Cx43 gap junction uncoupling prevents high glucose-induced apoptosis and reduces excess cell monolayer permeability in retinal vascular endothelial cells. *Exp. Eye Res.* 173 85–90. 10.1016/j.exer.2018.05.003 29750972

[B87] Kim K. J. Ramiro Diaz J. Iddings J. A. Filosa J. A. (2016). Vasculo-neuronal coupling: Retrograde vascular communication to brain neurons. *J. Neurosci.* 36 12624–12639. 10.1523/JNEUROSCI.1300-16.2016 27821575 PMC5157107

[B88] Kisler K. Nelson A. R. Montagne A. Zlokovic B. V. (2017). Cerebral blood flow regulation and neurovascular dysfunction in Alzheimer disease. *Nat. Rev. Neurosci.* 18 419–434. 28515434 10.1038/nrn.2017.48 PMC5759779

[B89] Kiss T. Nyúl-Tóth Á Balasubramanian P. Tarantini S. Ahire C. DelFavero J. (2020b). Single-cell RNA sequencing identifies senescent cerebromicrovascular endothelial cells in the aged mouse brain. *GeroScience* 42 429–444. 10.1007/s11357-020-00177-1 32236824 PMC7205992

[B90] Kiss T. Nyúl-Tóth Á Balasubramanian P. Tarantini S. Ahire C. Yabluchanskiy A. (2020a). Nicotinamide mononucleotide (NMN) supplementation promotes neurovascular rejuvenation in aged mice: Transcriptional footprint of SIRT1 activation, mitochondrial protection, anti-inflammatory, and anti-apoptotic effects. *GeroScience* 42 527–546. 10.1007/s11357-020-00165-5 32056076 PMC7206476

[B91] Kocharyan A. Fernandes P. Tong X.-K. Vaucher E. Hamel E. (2008). Specific subtypes of cortical GABA interneurons contribute to the neurovascular coupling response to basal forebrain stimulation. *J. Cereb. Blood Flow Metab.* 28 221–231. 10.1038/sj.jcbfm.9600558 17895909

[B92] Koekkoek P. S. Kappelle L. J. Van Den Berg E. Rutten G. E. H. M. Biessels G. J. (2015). Cognitive function in patients with diabetes mellitus: Guidance for daily care. *Lancet Neurol.* 14 329–340. 25728442 10.1016/S1474-4422(14)70249-2

[B93] Kovacs-Oller T. Ivanova E. Bianchimano P. Sagdullaev B. T. (2020). The pericyte connectome: Spatial precision of neurovascular coupling is driven by selective connectivity maps of pericytes and endothelial cells and is disrupted in diabetes. *Cell Discov.* 6:39. 10.1038/s41421-020-0180-0 32566247 PMC7296038

[B94] Kuzkaya N. Weissmann N. Harrison D. G. Dikalov S. (2003). Interactions of peroxynitrite, tetrahydrobiopterin, ascorbic acid, and thiols. *J. Biol. Chem.* 278 22546–22554. 12692136 10.1074/jbc.M302227200

[B95] Lecrux C. Sandoe C. Neupane S. Kropf P. Toussay X. Tong X. (2017). Impact of altered cholinergic tones on the neurovascular coupling response to whisker stimulation. *J. Neurosci.* 37 1518–1531. 10.1523/JNEUROSCI.1784-16.2016 28069927 PMC6705676

[B96] Lee D. Jeong G. (2016). Butein provides neuroprotective and anti-neuroinflammatory effects through Nrf2/ARE-dependent haem oxygenase 1 expression by activating the PI3K/Akt pathway. *Br. J. Pharmacol.* 173 2894–2909. 10.1111/bph.13569 27465039 PMC5055139

[B97] Li C. Yang Y. (2023). Advancements in the study of inward rectifying potassium channels on vascular cells. *Channels* 17:2237303. 10.1080/19336950.2023.2237303 37463317 PMC10355679

[B98] Li H. Ren J. Li Y. Wu Q. Wei J. (2023). Oxidative stress: The nexus of obesity and cognitive dysfunction in diabetes. *Front. Endocrinol.* 14:1134025. 10.3389/fendo.2023.1134025 37077347 PMC10107409

[B99] Li L. Tong X. Hosseini Kahnouei M. Vallerand D. Hamel E. Girouard H. (2021). Impaired hippocampal neurovascular coupling in a mouse model of Alzheimer’s disease. *Front. Physiol.* 12:715446. 10.3389/fphys.2021.715446 34475828 PMC8406685

[B100] Li S. Cui N. Yang Y. Trower T. Wei Y. Wu Y. (2015). Impairment of the vascular K ATP channel imposes fatal susceptibility to experimental diabetes due to multi-organ injuries. *J. Cell. Physiol.* 230 2915–2926. 10.1002/jcp.25003 25825210

[B101] Li W. Choudhury G. Winters A. Prah J. Lin W. Liu R. (2018). Hyperglycemia alters astrocyte metabolism and inhibits astrocyte proliferation. *Aging Dis.* 9:674. 10.14336/AD.2017.1208 30090655 PMC6065301

[B102] Li Y. Li Q. Pan C. Yan L. Hu B. Liu Y. (2018). Bushen Huoxue attenuates diabetes-induced cognitive impairment by improvement of cerebral microcirculation: Involvement of RhoA/ROCK/moesin and Src signaling pathways. *Front. Physiol.* 9:527. 10.3389/fphys.2018.00527 29867568 PMC5962779

[B103] Lind B. L. Brazhe A. R. Jessen S. B. Tan F. C. C. Lauritzen M. J. (2013). Rapid stimulus-evoked astrocyte Ca 2+ elevations and hemodynamic responses in mouse somatosensory cortex in vivo. *Proc. Natl. Acad. Sci. U.S.A.* 110 E4678–E4687. 10.1073/pnas.1310065110 24218625 PMC3845114

[B104] Little K. Llorián-Salvador M. Scullion S. Hernández C. Simó-Servat O. Del Marco A. (2022). Common pathways in dementia and diabetic retinopathy: Understanding the mechanisms of diabetes-related cognitive decline. *Trends Endocrinol. Metab.* 33 50–71. 10.1016/j.tem.2021.10.008 34794851

[B105] Liu J. Yang X. Li Y. Xu H. Ren J. Zhou P. (2022). Cerebral blood flow alterations in type 2 diabetes mellitus: A systematic review and meta-analysis of arterial spin labeling studies. *Front. Aging Neurosci.* 14:847218. 10.3389/fnagi.2022.847218 35250549 PMC8888831

[B106] Liu Y. (2002). The coronary circulation in diabetes influence of reactive oxygen species on K+ channel-mediated vasodilation. *Gen. Pharmacol. Vasc. Syst.* 38 43–49. 10.1016/s1537-1891(02)00125-8 12378822

[B107] Liu Y. Jiang Y. Du W. Gao B. Gao J. Hu S. (2024). White matter microstructure alterations in type 2 diabetes mellitus and its correlation with cerebral small vessel disease and cognitive performance. *Sci. Rep.* 14:270. 10.1038/s41598-023-50768-z 38167604 PMC10762026

[B108] Liu Y. Zhang Y. Zheng X. Fang T. Yang X. Luo X. (2018). Galantamine improves cognition, hippocampal inflammation, and synaptic plasticity impairments induced by lipopolysaccharide in mice. *J. Neuroinflammation* 15:112. 10.1186/s12974-018-1141-5 29669582 PMC5907415

[B109] Longden T. A. Nelson M. T. (2015). Vascular inward rectifier K + channels as external K + sensors in the control of cerebral blood flow. *Microcirculation* 22 183–196. 10.1111/micc.12190 25641345 PMC4404517

[B110] Longden T. A. Dabertrand F. Koide M. Gonzales A. Tykocki N. Brayden J. (2017). Capillary K+-sensing initiates retrograde hyperpolarization to increase local cerebral blood flow. *Nat. Neurosci.* 20 717–726. 28319610 10.1038/nn.4533 PMC5404963

[B111] Lorenzo P. I. Martin Vazquez E. López-Noriega L. Fuente-Martín E. Mellado-Gil J. Franco J. (2021). The metabesity factor HMG20A potentiates astrocyte survival and reactive astrogliosis preserving neuronal integrity. *Theranostics* 11 6983–7004. 10.7150/thno.57237 34093866 PMC8171100

[B112] Lourenço C. F. Laranjinha J. (2021). Nitric oxide pathways in neurovascular coupling under normal and stress conditions in the brain: Strategies to rescue aberrant coupling and improve cerebral blood flow. *Front. Physiol.* 12:729201. 10.3389/fphys.2021.729201 34744769 PMC8569710

[B113] Lourenço C. F. Ledo A. Barbosa R. M. Laranjinha J. (2017). Neurovascular-neuroenergetic coupling axis in the brain: Master regulation by nitric oxide and consequences in aging and neurodegeneration. *Free Radic. Biol. Med.* 108 668–682. 10.1016/j.freeradbiomed.2017.04.026 28435052

[B114] Ly H. Verma N. Wu F. Liu M. Saatman K. Nelson P. (2017). Brain microvascular injury and white matter disease provoked by diabetes-associated hyperamylinemia. *Ann. Neurol.* 82 208–222. 10.1002/ana.24992 28696548 PMC5568970

[B115] Ma M. Hasegawa Y. Koibuchi N. Toyama K. Uekawa K. Nakagawa T. (2015). DPP-4 inhibition with linagliptin ameliorates cognitive impairment and brain atrophy induced by transient cerebral ischemia in type 2 diabetic mice. *Cardiovasc. Diabetol.* 14:54. 10.1186/s12933-015-0218-z 25986579 PMC4458052

[B116] Ma S. Wang J. Wang Y. Dai X. Xu F. Gao X. (2018). Diabetes mellitus impairs white matter repair and long-term functional deficits after cerebral ischemia. *Stroke* 49 2453–2463. 10.1161/STROKEAHA.118.021452 30355111 PMC6205761

[B117] Magariños A. M. McEwen B. S. (2000). Experimental diabetes in rats causes hippocampal dendritic and synaptic reorganization and increased glucocorticoid reactivity to stress. *Proc. Natl. Acad. Sci. U.S.A.* 97 11056–11061. 10.1073/pnas.97.20.11056 11005876 PMC27147

[B118] Maurer M. H. Geomor H. K. Bürgers H. F. Schelshorn D. W. Kuschinsky W. (2006). Adult neural stem cells express glucose transporters GLUT1 and GLUT3 and regulate GLUT3 expression. *FEBS Lett.* 580 4430–4434. 10.1016/j.febslet.2006.07.012 16854415

[B119] Mauricio D. Gratacòs M. Franch-Nadal J. (2023). Diabetic microvascular disease in non-classical beds: The hidden impact beyond the retina, the kidney, and the peripheral nerves. *Cardiovasc. Diabetol.* 22:314. 10.1186/s12933-023-02056-3 37968679 PMC10652502

[B120] Mayhan W. G. Mayhan J. F. Sun H. Patel K. P. (2004). In vivo properties of potassium channels in cerebral blood vessels during diabetes mellitus. *Microcirculation* 11 605–613. 10.1080/10739680490503410 15513870

[B121] Mazaika P. K. Marzelli M. Tong G. Foland-Ross L. Buckingham B. Aye T. (2020). Functional near-infrared spectroscopy detects increased activation of the brain frontal-parietal network in youth with type 1 diabetes. *Pediatr. Diabetes* 21 515–523. 10.1111/pedi.12992 32003523

[B122] McConnell H. L. Kersch C. N. Woltjer R. L. Neuwelt E. A. (2017). The translational significance of the neurovascular unit. *J. Biol. Chem.* 292 762–770. 27920202 10.1074/jbc.R116.760215 PMC5247651

[B123] McManus R. Ioussoufovitch S. Froats E. St Lawrence K. Van Uum S. Diop M. (2020). Dynamic response of cerebral blood flow to insulin-induced hypoglycemia. *Sci. Rep.* 10:21300. 10.1038/s41598-020-77626-6 33277531 PMC7718270

[B124] Mellado-Gil J. M. Fuente-Martín E. Lorenzo P. Cobo-Vuilleumier N. López-Noriega L. Martín-Montalvo A. (2018). The type 2 diabetes-associated HMG20A gene is mandatory for islet beta cell functional maturity. *Cell Death Dis.* 9:279. 10.1038/s41419-018-0272-z 29449530 PMC5833347

[B125] Meng F. Fu J. Zhang L. Guo M. Zhuang P. Yin Q. (2023). Function and therapeutic value of astrocytes in diabetic cognitive impairment. *Neurochem. Int.* 169:105591. 10.1016/j.neuint.2023.105591 37543309

[B126] Misumi Y. Yamato T. Obata T. Aomine M. (2008). Effects of ion channel blockers on basal hippocampal monoamine levels in freely moving diabetic and non-diabetic rats. *Int. J. Neurosci.* 118 761–780. 10.1080/00207450600941106 18465423

[B127] Mogi M. Horiuchi M. (2011). Neurovascular coupling in cognitive impairment associated with diabetes mellitus. *Circ. J.* 75 1042–1048. 21441696 10.1253/circj.cj-11-0121

[B128] Monteiro A. Castro P. Pereira G. Ferreira C. Sorond F. Milstead A. (2021). Neurovascular coupling is impaired in hypertensive and diabetic subjects without symptomatic cerebrovascular disease. *Front. Aging Neurosci.* 13:728007. 10.3389/fnagi.2021.728007 34690741 PMC8526560

[B129] Moon D.-O. (2023). NADPH dynamics: Linking insulin resistance and β-Cells ferroptosis in diabetes mellitus. *Int. J. Mol. Sci.* 25:342. 10.3390/ijms25010342 PMC10779351 38203517

[B130] Mugisho O. Green C. Zhang J. Binz N. Acosta M. Rakoczy E. (2017). Immunohistochemical characterization of connexin43 expression in a mouse model of diabetic retinopathy and in human donor retinas. *Int. J. Mol. Sci.* 18:2567. 10.3390/ijms18122567 29186067 PMC5751170

[B131] Muhammad T. Ikram M. Ullah R. Rehman S. Kim M. (2019). Hesperetin, a citrus flavonoid, attenuates LPS-induced neuroinflammation, apoptosis and memory impairments by modulating TLR4/NF-κB signaling. *Nutrients* 11:648. 10.3390/nu11030648 PMC6471991 30884890

[B132] Nardin P. Zanotto C. Hansen F. Batassini C. Gasparin M. Sesterheim P. (2016). Peripheral levels of ages and astrocyte alterations in the hippocampus of STZ-diabetic rats. *Neurochem. Res.* 41 2006–2016. 10.1007/s11064-016-1912-2 27084774

[B133] Ni M.-H. Li Z. Sun Q. Yu Y. Yang Y. Hu B. (2023). Neurovascular decoupling measured with quantitative susceptibility mapping is associated with cognitive decline in patients with type 2 diabetes. *Cereb. Cortex* 33 5336–5346. 10.1093/cercor/bhac422 36310091

[B134] Nieves-Cintrón M. Syed A. Buonarati O. Rigor R. Nystoriak M. Ghosh D. (2017). Impaired BKCa channel function in native vascular smooth muscle from humans with type 2 diabetes. *Sci. Rep.* 7:14058. 10.1038/s41598-017-14565-9 29070899 PMC5656614

[B135] Nimlamool W. Andrews R. M. K. Falk M. M. (2015). Connexin43 phosphorylation by PKC and MAPK signals VEGF-mediated gap junction internalization. *Mol. Biol. Cell* 26 2755–2768. 10.1091/mbc.E14-06-1105 26063728 PMC4571336

[B136] Nippert A. R. Chiang P.-P. Newman E. A. (2023). Whisker-evoked neurovascular coupling is preserved during hypoglycemia in mouse cortical arterioles and capillaries. *J. Cereb. Blood Flow Metab.* 44 155–168. 10.1177/0271678X231201241 37728791 PMC10993878

[B137] Nippert A. R. Chiang P.-P. Del Franco A. P. Newman E. A. (2022). Astrocyte regulation of cerebral blood flow during hypoglycemia. *J. Cereb. Blood Flow Metab.* 42 1534–1546. 35296178 10.1177/0271678X221089091 PMC9274859

[B138] Nizar K. Uhlirova H. Tian P. Saisan P. Cheng Q. Reznichenko L. (2013). In vivo stimulus-induced vasodilation occurs without IP 3 receptor activation and may precede astrocytic calcium increase. *J. Neurosci.* 33 8411–8422. 10.1523/JNEUROSCI.3285-12.2013 23658179 PMC3712855

[B139] Noce B. Di Bello E. Fioravanti R. Mai A. (2023). LSD1 inhibitors for cancer treatment: Focus on multi-target agents and compounds in clinical trials. *Front. Pharmacol.* 14:1120911. 10.3389/fphar.2023.1120911 36817147 PMC9932783

[B140] Novak V. Last D. Alsop D. Abduljalil A. Hu K. Lepicovsky L. (2006). Cerebral blood flow velocity and periventricular white matter hyperintensities in type 2 diabetes. *Diabetes Care* 29 1529–1534. 16801574 10.2337/dc06-0261 PMC1978169

[B141] Ohara T. Furuta Y. Hirabayashi N. Hata J. Hirakawa Y. Honda T. (2020). Elevated serum glycated albumin and glycated albumin: Hemoglobin A 1c ratio were associated with hippocampal atrophy in a general elderly population of Japanese: The Hisayama study. *J. Diabetes Investig.* 11 971–979. 10.1111/jdi.13220 PMC7378434 31999889

[B142] Okayasu M. Inukai T. Tanaka D. Tsumura K. Shintaki R. Takeda M. (2023). The Stroop effect involves an excitatory–inhibitory fronto-cerebellar loop. *Nat. Commun.* 14:27. 10.1038/s41467-022-35397-w 36631460 PMC9834394

[B143] Otsu Y. Couchman K. Lyons D. Collot M. Agarwal A. Mallet J. (2015). Calcium dynamics in astrocyte processes during neurovascular coupling. *Nat. Neurosci.* 18 210–218. 25531572 10.1038/nn.3906 PMC4651918

[B144] Ott C. Jacobs K. Haucke E. Navarrete Santos A. Grune T. Simm A. (2014). Role of advanced glycation end products in cellular signaling. *Redox Biol.* 2 411–429. 24624331 10.1016/j.redox.2013.12.016 PMC3949097

[B145] Ottolini M. Hong K. Sonkusare S. K. (2019). Calcium signals that determine vascular resistance. *WIREs Syst. Biol. Med.* 11:e1448. 10.1002/wsbm.1448 PMC6688910 30884210

[B146] Pan D. Xu L. Guo M. (2022). The role of protein kinase C in diabetic microvascular complications. *Front. Endocrinol.* 13:973058. 10.3389/fendo.2022.973058 36060954 PMC9433088

[B147] Parsamanesh N. Asghari A. Sardari S. Tasbandi A. Jamialahmadi T. Xu S. (2021). Resveratrol and endothelial function: A literature review. *Pharmacol. Res.* 170:105725. 10.1016/j.phrs.2021.105725 34119624

[B148] Paulson O. B. Newman E. A. (1987). Does the release of potassium from astrocyte endfeet regulate cerebral blood flow? *Science* 237 896–898. 3616619 10.1126/science.3616619 PMC2505270

[B149] Petravicz J. Fiacco T. A. McCarthy K. D. (2008). Loss of IP 3 receptor-dependent Ca 2+ increases in hippocampal astrocytes does not affect baseline CA1 pyramidal neuron synaptic activity. *J. Neurosci.* 28 4967–4973. 10.1523/JNEUROSCI.5572-07.2008 18463250 PMC2709811

[B150] Prattichizzo F. De Nigris V. Mancuso E. Spiga R. Giuliani A. Matacchione G. (2018). Short-term sustained hyperglycaemia fosters an archetypal senescence-associated secretory phenotype in endothelial cells and macrophages. *Redox Biol.* 15 170–181. 10.1016/j.redox.2017.12.001 29253812 PMC5735298

[B151] Price T. O. Sheibani N. Shah G. N. (2017). Regulation of high glucose-induced apoptosis of brain pericytes by mitochondrial CA VA: A specific target for prevention of diabetic cerebrovascular pathology. *Biochim. Biophys. Acta BBA Mol. Basis Dis.* 1863 929–935. 10.1016/j.bbadis.2017.01.025 28131914 PMC5328884

[B152] Raut S. Bhalerao A. Powers M. Gonzalez M. Mancuso S. Cucullo L. (2023). Hypometabolism, Alzheimer’s disease, and possible therapeutic targets: An overview. *Cells* 12:2019. 10.3390/cells12162019 37626828 PMC10453773

[B153] Rezende F. Moll F. Walter M. Helfinger V. Hahner F. Janetzko P. (2018). The NADPH organizers NoxO1 and p47phox are both mediators of diabetes-induced vascular dysfunction in mice. *Redox Biol.* 15 12–21. 29195137 10.1016/j.redox.2017.11.014 PMC5723277

[B154] Rizzo M. R. Di Meo I. Polito R. Auriemma M. Gambardella A. di Mauro G. (2022). Cognitive impairment and type 2 diabetes mellitus: Focus of SGLT2 inhibitors treatment. *Pharmacol. Res.* 176:106062. 10.1016/j.phrs.2022.106062 35017046

[B155] Robb J. L. Morrissey N. Weightman Potter P. Smithers H. Beall C. Ellacott K. (2020). Immunometabolic Changes in glia – a potential role in the pathophysiology of obesity and diabetes. *Neuroscience* 447 167–181. 10.1016/j.neuroscience.2019.10.021 31765625 PMC7567742

[B156] Roberts R. O. Knopman D. Geda Y. Cha R. Pankratz V. Baertlein L. (2014). Association of diabetes with amnestic and nonamnestic mild cognitive impairment. *Alzheimers Dement.* 10 18–26. 23562428 10.1016/j.jalz.2013.01.001 PMC3830601

[B157] Robinson M. B. Jackson J. G. (2016). Astroglial glutamate transporters coordinate excitatory signaling and brain energetics. *Neurochem. Int.* 98 56–71. 10.1016/j.neuint.2016.03.014 27013346 PMC4969184

[B158] Rom S. Heldt N. Gajghate S. Seliga A. Reichenbach N. Persidsky Y. (2020). Hyperglycemia and advanced glycation end products disrupt BBB and promote occludin and claudin-5 protein secretion on extracellular microvesicles. *Sci. Rep.* 10:7274. 10.1038/s41598-020-64349-x PMC7190636 32350344

[B159] Rom S. Zuluaga-Ramirez V. Reichenbach N. Erickson M. Winfield M. Gajghate S. (2018). Secoisolariciresinol diglucoside is a blood-brain barrier protective and anti-inflammatory agent: Implications for neuroinflammation. *J. Neuroinflammation* 15:25. 10.1186/s12974-018-1065-0 29373982 PMC5787274

[B160] Rungta R. L. Charpak S. (2016). Astrocyte endfeet march to the beat of different vessels. *Nat. Neurosci.* 19 1539–1541. 10.1038/nn.4446 27898083

[B161] Sadanand S. Balachandar R. Bharath S. (2016). Memory and executive functions in persons with type 2 diabetes: A meta-analysis: Type 2 Diabetes and Cognition. *Diabetes Metab. Res. Rev.* 32 132–142. 25963303 10.1002/dmrr.2664

[B162] Sakib M. N. Ramezan R. Hall P. A. (2023). Diabetes status and cognitive function in middle-aged and older adults in the Canadian longitudinal study on aging. *Front. Endocrinol.* 14:1293988. 10.3389/fendo.2023.1293988 38107512 PMC10722407

[B163] Salamero O. Montesinos P. Willekens C. Pérez-Simón J. Pigneux A. Récher C. (2020). First-in-human phase I study of iadademstat (ORY-1001): A first-in-class lysine-specific histone demethylase 1A inhibitor, in relapsed or refractory acute myeloid leukemia. *J. Clin. Oncol.* 38 4260–4273. 33052756 10.1200/JCO.19.03250 PMC7768337

[B164] Sampath C. Raju A. V. Freeman M. L. Srinivasan S. Gangula P. R. (2022). Nrf2 attenuates hyperglycemia-induced nNOS impairment in adult mouse primary enteric neuronal crest cells and normalizes stomach function. *Am. J. Physiol. Gastrointest. Liver Physiol.* 322 G368–G382. 10.1152/ajpgi.00323.2021 35084215 PMC8897013

[B165] Sampath C. Sprouse J. C. Freeman M. L. Gangula P. R. (2019). Activation of Nrf2 attenuates delayed gastric emptying in obesity induced diabetic (T2DM) female mice. *Free Radic. Biol. Med.* 135 132–143. 30831189 10.1016/j.freeradbiomed.2019.02.029 PMC6738571

[B166] Sanahuja J. Alonso N. Diez J. Ortega E. Rubinat E. Traveset A. (2016). Increased burden of cerebral small vessel disease in patients with type 2 diabetes and retinopathy. *Diabetes Care* 39 1614–1620. 27281772 10.2337/dc15-2671

[B167] Satoh E. Takahashi A. (2008). Experimental diabetes enhances Ca2+ mobilization and glutamate exocytosis in cerebral synaptosomes from mice. *Diabetes Res. Clin. Pract.* 81 e14–e17. 10.1016/j.diabres.2008.04.017 18508149

[B168] Scamarcia P. G. Agosta F. Spinelli E. Basaia S. Stojkoviæ T. Stankovic I. (2022). Longitudinal white matter damage evolution in Parkinson’s disease. *Mov. Disord.* 37 315–324. 10.1002/mds.28864 34806799

[B169] Schaeffer S. Iadecola C. (2021). Revisiting the neurovascular unit. *Nat. Neurosci.* 24 1198–1209. 34354283 10.1038/s41593-021-00904-7 PMC9462551

[B170] Schneider A. L. C. Selvin E. Sharrett A. Griswold M. Coresh J. Jack C. Jr. (2017). Diabetes, prediabetes, and brain volumes and subclinical cerebrovascular disease on MRI: The atherosclerosis risk in communities neurocognitive study (ARIC-NCS). *Diabetes Care* 40 1514–1521. 10.2337/dc17-1185 28916531 PMC5652590

[B171] Schulz K. Sydekum E. Krueppel R. Engelbrecht C. Schlegel F. Schröter A. (2012). Simultaneous BOLD fMRI and fiber-optic calcium recording in rat neocortex. *Nat. Methods* 9 597–602. 22561989 10.1038/nmeth.2013

[B172] Schummers J. Yu H. Sur M. (2008). Tuned responses of astrocytes and their influence on hemodynamic signals in the visual cortex. *Science* 320 1638–1643. 18566287 10.1126/science.1156120

[B173] Secnik J. Xu H. Schwertner E. Hammar N. Alvarsson M. Winblad B. (2021). The association of antidiabetic medications and mini-mental state examination scores in patients with diabetes and dementia. *Alzheimers Res. Ther.* 13:197. 10.1186/s13195-021-00934-0 PMC8641148 34857046

[B174] Sedovy M. W. Leng X. Leaf M. Iqbal F. Payne L. Chappell J. (2023). Connexin 43 across the vasculature: Gap junctions and beyond. *J. Vasc. Res.* 60 101–113. 10.1159/000527469 36513042 PMC11073551

[B175] Seifert T. Secher N. H. (2011). Sympathetic influence on cerebral blood flow and metabolism during exercise in humans. *Prog. Neurobiol.* 95 406–426. 21963551 10.1016/j.pneurobio.2011.09.008

[B176] Senthil K. K. J. Gokila V. M. Wang S.-Y. (2017). Activation of Nrf2-mediated anti-oxidant genes by antrodin C prevents hyperglycemia-induced senescence and apoptosis in human endothelial cells. *Oncotarget* 8 96568–96587. 10.18632/oncotarget.19951 29228553 PMC5722505

[B177] Shah G. N. Morofuji Y. Banks W. A. Price T. O. (2013). High glucose-induced mitochondrial respiration and reactive oxygen species in mouse cerebral pericytes is reversed by pharmacological inhibition of mitochondrial carbonic anhydrases: Implications for cerebral microvascular disease in diabetes. *Biochem. Biophys. Res. Commun.* 440 354–358. 10.1016/j.bbrc.2013.09.086 24076121 PMC3875343

[B178] Shaw K. Bell L. Boyd K. Grijseels D. Clarke D. Bonnar O. (2021). Neurovascular coupling and oxygenation are decreased in hippocampus compared to neocortex because of microvascular differences. *Nat. Commun.* 12:3190. 10.1038/s41467-021-23508-y PMC8160329 34045465

[B179] Shekhar S. Wang S. Mims P. Gonzalez-Fernandez E. Zhang C. He X. (2017). Impaired cerebral autoregulation-a common neurovascular pathway in diabetes may play a critical role in diabetes-related Alzheimer’s disease. *Curr. Res. Diabetes Obes. J.* 2:555587. 28825056 PMC5559201

[B180] Shi S. Yin H. Li J. Wang L. Wang W. Wang X. (2020). Studies of pathology and pharmacology of diabetic encephalopathy with KK-Ay mouse model. *CNS Neurosci. Ther.* 26 332–342. 10.1111/cns.13201 31401815 PMC7052806

[B181] Shibata M. Nakaizumi A. Puro D. G. (2019). Electrotonic transmission in the retinal vasculature: Inhibitory role of the diabetes/ VEGF / APKC pathway. *Physiol. Rep.* 7:e14095. 10.14814/phy2.14095 31087517 PMC6513771

[B182] Sinclair A. Abdelhafiz A. (2020). Cognitive Dysfunction in older adults with type 2 diabetes. *Clin. Geriatr. Med.* 36 407–417. 32586471 10.1016/j.cger.2020.04.002

[B183] Stackhouse T. L. Mishra A. (2021). Neurovascular coupling in development and disease: Focus on astrocytes. *Front. Cell Dev. Biol.* 9:702832. 10.3389/fcell.2021.702832 34327206 PMC8313501

[B184] Suenkel B. Valente S. Zwergel C. Weiss S. Di Bello E. Fioravanti R. (2022). Potent and specific activators for mitochondrial sirtuins Sirt3 and Sirt5. *J. Med. Chem.* 65 14015–14031. 10.1021/acs.jmedchem.2c01215 36228194 PMC9653166

[B185] Sweeney M. D. Ayyadurai S. Zlokovic B. V. (2016). Pericytes of the neurovascular unit: Key functions and signaling pathways. *Nat. Neurosci.* 19 771–783. 27227366 10.1038/nn.4288 PMC5745011

[B186] Tarantini S. Hertelendy P. Tucsek Z. Valcarcel-Ares M. Smith N. Menyhart A. (2015). Pharmacologically-induced neurovascular uncoupling is associated with cognitive impairment in mice. *J. Cereb. Blood Flow Metab.* 35 1871–1881. 26174328 10.1038/jcbfm.2015.162 PMC4635246

[B187] Tiehuis A. M. Vincken K. van den Berg E. Hendrikse J. Manschot S. Mali W. (2008). Cerebral perfusion in relation to cognitive function and type 2 diabetes. *Diabetologia* 51 1321–1326. 18488188 10.1007/s00125-008-1041-9 PMC2440938

[B188] Tien T. Barrette K. F. Chronopoulos A. Roy S. (2013). Effects of high glucose-induced Cx43 downregulation on occludin and ZO-1 expression and tight junction barrier function in retinal endothelial cells. *Investig. Opthalmol. Vis. Sci.* 54:6518. 10.1167/iovs.13-11763 24008412 PMC3790390

[B189] Torabi N. Noursadeghi E. Shayanfar F. Nazari M. Fahanik-Babaei J. Saghiri R. (2021). Intranasal insulin improves the structure–function of the brain mitochondrial ATP–sensitive Ca2+ activated potassium channel and respiratory chain activities under diabetic conditions. *Biochim. Biophys. Acta BBA Mol. Basis Dis.* 1867:166075. 10.1016/j.bbadis.2021.166075 33444710

[B190] Toth P. Tarantini S. Ashpole N. Tucsek Z. Milne G. Valcarcel-Ares N. (2015a). IGF -1 deficiency impairs neurovascular coupling in mice: Implications for cerebromicrovascular aging. *Aging Cell* 14 1034–1044. 10.1111/acel.12372 26172407 PMC4693458

[B191] Toth P. Tarantini S. Davila A. Valcarcel-Ares M. Tucsek Z. Varamini B. (2015b). Purinergic glio-endothelial coupling during neuronal activity: Role of P2Y 1 receptors and eNOS in functional hyperemia in the mouse somatosensory cortex. *Am. J. Physiol. Heart Circ. Physiol.* 309 H1837–H1845. 10.1152/ajpheart.00463.2015 26453330 PMC4698379

[B192] Tu W. Song M. Fan X. (2023). Does resveratrol improve cognition in humans? A scientometric study to an in-depth review. *CNS Neurosci. Ther.* 29 2413–2429. 10.1111/cns.14276 37248634 PMC10401104

[B193] Turner D. A. (2021). Contrasting metabolic insufficiency in aging and dementia. *Aging Dis.* 12 1081. 10.14336/AD.2021.0104 PMC8219502 34221551

[B194] Van Den Berg M. Toen D. Verhoye M. Keliris G. A. (2023). Alterations in theta-gamma coupling and sharp wave-ripple, signs of prodromal hippocampal network impairment in the TgF344-AD rat model. *Front. Aging Neurosci.* 15:1081058. 10.3389/fnagi.2023.1081058 37032829 PMC10075364

[B195] Venkat P. Chopp M. Chen J. (2016). New insights into coupling and uncoupling of cerebral blood flow and metabolism in the brain. *Croat. Med. J.* 57 223–228. 27374823 10.3325/cmj.2016.57.223 PMC4937223

[B196] Vetri F. Chavez R. Xu H.-L. Paisansathan C. Pelligrino D. A. (2013). Complex modulation of the expression of PKC isoforms in the rat brain during chronic type 1 diabetes mellitus. *Brain Res.* 1490 202–209. 10.1016/j.brainres.2012.10.032 23103504 PMC3529837

[B197] Vetri F. Qi M. Xu H. Oberholzer J. Paisansathan C. (2017). Impairment of neurovascular coupling in type 1 diabetes mellitus in rats is prevented by pancreatic islet transplantation and reversed by a semi-selective PKC inhibitor. *Brain Res.* 1655 48–54. 10.1016/j.brainres.2016.11.012 27865779 PMC5195876

[B198] Vetri F. Xu H. Paisansathan C. Pelligrino D. A. (2012). Impairment of neurovascular coupling in type 1 diabetes mellitus in rats is linked to PKC modulation of BK Ca and Kir channels. *Am. J. Physiol. Heart Circ. Physiol.* 302 H1274–H1284. 10.1152/ajpheart.01067.2011 22268114 PMC3311480

[B199] Wallerath T. Deckert G. Ternes T. Anderson H. Li H. Witte K. (2002). Resveratrol, a polyphenolic phytoalexin present in red wine, enhances expression and activity of endothelial nitric oxide synthase. *Circulation* 106 1652–1658. 10.1161/01.cir.0000029925.18593.5c 12270858

[B200] Wang H. Tang W. Zhao Y. (2023). Acute effects of different exercise forms on executive function and the mechanism of cerebral hemodynamics in hospitalized T2DM patients: A within-subject study. *Front. Public Health* 11:1165892. 10.3389/fpubh.2023.1165892 37333536 PMC10270376

[B201] Wang Y.-C. Wang L. Shao Y. Weng S. Yang X. Zhong Y. (2023). Exendin-4 promotes retinal ganglion cell survival and function by inhibiting calcium channels in experimental diabetes. *iScience* 26:107680. 10.1016/j.isci.2023.107680 37680468 PMC10481356

[B202] Wang J. Zhao C. Wei J. Li C. Zhang X. Liang Y. (2022). Individual prediction and classification of cognitive impairment in patients with white matter lesions based on gray matter volume. *Ann. Transl. Med.* 10 246–246. 35402600 10.21037/atm-21-3571 PMC8987882

[B203] Wang X.-P. Ye P. Lv J. Zhou L. Qian Z. Huang Y. (2019). Expression changes of NMDA and AMPA receptor subunits in the hippocampus in rats with diabetes induced by streptozotocin coupled with memory impairment. *Neurochem. Res.* 44 978–993. 10.1007/s11064-019-02733-4 30747310

[B204] Wang Y. Sun L. He G. Gang X. Zhao X. Wang G. (2021). Cerebral perfusion alterations in type 2 diabetes mellitus – a systematic review. *Front. Neuroendocrinol.* 62:100916. 10.1016/j.yfrne.2021.100916 33957174

[B205] Wang Y. Zhang H. Su X. Deng X. Yuan B. Zhang W. (2010). Experimental diabetes mellitus down-regulates large-conductance Ca2+- activated K+ channels in cerebral artery smooth muscle and alters functional conductance. *Curr. Neurovasc. Res.* 7 75–84. 10.2174/156720210791184925 20334613

[B206] Wells J. A. Christie I. Hosford P. Huckstepp R. Angelova P. Vihko P. (2015). A critical role for purinergic signalling in the mechanisms underlying generation of BOLD fMRI responses. *J. Neurosci.* 35 5284–5292. 10.1523/JNEUROSCI.3787-14.2015 25834053 PMC4381001

[B207] Wong R. Raederstorff D. Howe P. (2016). Acute resveratrol consumption improves neurovascular coupling capacity in adults with type 2 diabetes mellitus. *Nutrients* 8:425. 10.3390/nu8070425 27420093 PMC4963901

[B208] Wu G. Meininger C. J. (2009). Nitric oxide and vascular insulin resistance. *BioFactors* 35 21–27. 19319842 10.1002/biof.3

[B209] Wu L. Chen Y. Wang C. Tang Y. Huang H. Kang X. (2019). Hydrogen sulfide inhibits high glucose-induced neuronal senescence by improving autophagic flux via up-regulation of SIRT1. *Front. Mol. Neurosci.* 12:194. 10.3389/fnmol.2019.00194 31481873 PMC6710442

[B210] Xiang Q. Tao J. Dong S. Liu X. Yang L. Liu L. (2024). Heterogeneity and synaptic plasticity analysis of hippocampus based on db-/- mice induced diabetic encephalopathy. *Psychoneuroendocrinology* 159:106412. 10.1016/j.psyneuen.2023.106412 37898037

[B211] Xiong W. MacColl Garfinkel A. E. Li Y. Benowitz L. I. Cepko C. L. (2015). NRF2 promotes neuronal survival in neurodegeneration and acute nerve damage. *J. Clin. Invest.* 125 1433–1445. 10.1172/JCI79735 25798616 PMC4396467

[B212] Xiong Y. Sui Y. Zhang S. Zhou X. Yang S. Fan Y. (2019). Brain microstructural alterations in type 2 diabetes: Diffusion kurtosis imaging provides added value to diffusion tensor imaging. *Eur. Radiol.* 29 1997–2008. 10.1007/s00330-018-5746-y 30338363

[B213] Yamagishi S. Nakamura N. Matsui T. (2017). Glycation and cardiovascular disease in diabetes: A perspective on the concept of metabolic memory. *J. Diabetes* 9 141–148. 10.1111/1753-0407.12475 27556881

[B214] Yamanaka T. Uchida Y. Sakurai K. Kato D. Mizuno M. Sato T. (2019). Anatomical links between white matter hyperintensity and medial temporal atrophy reveal impairment of executive functions. *Aging Dis.* 10:711. 10.14336/AD.2018.0929 31440378 PMC6675535

[B215] Yan C. Zhou Y. Chen Q. Luo Y. Zhang J. Huang H. (2020). Dysfunction of the neurovascular unit in diabetes-related neurodegeneration. *Biomed. Pharmacother.* 131:110656. 10.1016/j.biopha.2020.110656 32841897

[B216] Yan W. Pang M. Yu Y. Gou X. Si P. Zhawatibai A. (2019). The neuroprotection of liraglutide on diabetic cognitive deficits is associated with improved hippocampal synapses and inhibited neuronal apoptosis. *Life Sci.* 231:116566. 10.1016/j.lfs.2019.116566 31201846

[B217] Yang C.-M. Lin C.-C. Hsieh H.-L. (2017). High-glucose-derived oxidative stress-dependent heme oxygenase-1 expression from astrocytes contributes to the neuronal apoptosis. *Mol. Neurobiol.* 54 470–483. 10.1007/s12035-015-9666-4 26742524

[B218] Yang Y. Shi W. Chen X. Cui N. Konduru A. Shi Y. (2011). Molecular basis and structural insight of vascular KATP channel gating by S-glutathionylation. *J. Biol. Chem.* 286 9298–9307. 10.1074/jbc.M110.195123 21216949 PMC3059003

[B219] Yatomi Y. Tanaka R. Shimada Y. Yamashiro K. Liu M. Mitome-Mishima Y. (2015). Type 2 diabetes reduces the proliferation and survival of oligodendrocyte progenitor cells in ishchemic white matter lesions. *Neuroscience* 289 214–223. 10.1016/j.neuroscience.2014.12.054 25592431

[B220] Yoo D. Y. Yim H. Jung H. Nam S. Kim J. Choi J. (2016). Chronic type 2 diabetes reduces the integrity of the blood-brain barrier by reducing tight junction proteins in the hippocampus. *J. Vet. Med. Sci.* 78 957–962. 10.1292/jvms.15-0589 26876499 PMC4937155

[B221] Yu Y. Fu P. Yu Z. Xie M. Wang W. Luo X. (2018). NKCC1 inhibition attenuates chronic cerebral hypoperfusion-induced white matter lesions by enhancing progenitor cells of oligodendrocyte proliferation. *J. Mol. Neurosci.* 64 449–458. 10.1007/s12031-018-1043-0 29502291

[B222] Yu Y. Yan L. Sun Q. Hu B. Zhang J. Yang Y. (2019). Neurovascular decoupling in type 2 diabetes mellitus without mild cognitive impairment: Potential biomarker for early cognitive impairment. *Neuroimage* 200 644–658. 31252056 10.1016/j.neuroimage.2019.06.058

[B223] Zhang F.-X. Xu R.-S. (2018). Juglanin ameliorates LPS-induced neuroinflammation in animal models of Parkinson’s disease and cell culture via inactivating TLR4/NF-κB pathway. *Biomed. Pharmacother.* 97 1011–1019. 29136779 10.1016/j.biopha.2017.08.132

[B224] Zhang H. Roman R. J. Fan F. (2022). Hippocampus is more susceptible to hypoxic injury: Has the Rosetta stone of regional variation in neurovascular coupling been deciphered? *GeroScience* 44 127–130. 10.1007/s11357-021-00449-4 34453273 PMC8810993

[B225] Zhang Q. Liu J. Duan H. Li R. Peng W. Wu C. (2021). Activation of Nrf2/HO-1 signaling: An important molecular mechanism of herbal medicine in the treatment of atherosclerosis via the protection of vascular endothelial cells from oxidative stress. *J. Adv. Res.* 34 43–63. 10.1016/j.jare.2021.06.023 35024180 PMC8655139

[B226] Zhang W. Gao C. Qing Z. Zhang Z. Bi Y. Zeng W. (2021). Hippocampal subfields atrophy contribute more to cognitive impairment in middle-aged patients with type 2 diabetes rather than microvascular lesions. *Acta Diabetol.* 58 1023–1033. 10.1007/s00592-020-01670-x 33751221

[B227] Zhang Y. Zhang X. Ma G. Qin W. Yang J. Lin J. (2021). Neurovascular coupling alterations in type 2 diabetes: A 5-year longitudinal MRI study. *BMJ Open Diabetes Res. Care* 9:e001433. 10.1136/bmjdrc-2020-001433 33462074 PMC7816934

[B228] Zhou G.-Y. Yi Y. Jin L. Lin W. Fang P. Lin X. (2016). The protective effect of juglanin on fructose-induced hepatitis by inhibiting inflammation and apoptosis through TLR4 and JAK2/STAT3 signaling pathways in fructose-fed rats. *Biomed. Pharmacother.* 81 318–328. 10.1016/j.biopha.2016.04.013 27261609

[B229] Zhou S. Chen H. Wan Y. Zhang Q. Wei Y. Huang S. (2011). Repression of P66Shc expression by SIRT1 contributes to the prevention of hyperglycemia-induced endothelial dysfunction. *Circ. Res.* 109 639–648. 10.1161/CIRCRESAHA.111.243592 21778425

[B230] Zhou Y. Huang L. Zheng W. An J. Zhan Z. Wang L. (2018). Recurrent nonsevere hypoglycemia exacerbates imbalance of mitochondrial homeostasis leading to synapse injury and cognitive deficit in diabetes. *Am. J. Physiol. Endocrinol. Metab.* 315 E973–E986. 10.1152/ajpendo.00133.2018 29969317

[B231] Zwergel C. Aventaggiato M. Garbo S. Di Bello E. Fassari B. Noce B. (2023). Novel 1,4-dihydropyridines as specific binders and activators of SIRT3 impair cell viability and clonogenicity and downregulate hypoxia-induced targets in cancer cells. *J. Med. Chem.* 66 9622–9641. 10.1021/acs.jmedchem.3c00337 37439550 PMC10388363

